# Lenvatinib for effectively treating antiangiogenic drug-resistant nasopharyngeal carcinoma

**DOI:** 10.1038/s41419-022-05171-3

**Published:** 2022-08-19

**Authors:** Qi Sun, Yujie Wang, Hong Ji, Xiaoting Sun, Sisi Xie, Longtian Chen, Sen Li, Weifan Zeng, Ruibo Chen, Qi Tang, Ji Zuo, Likun Hou, Kayoko Hosaka, Yongtian Lu, Ying Liu, Ying Ye, Yunlong Yang

**Affiliations:** 1 Department of Cellular and Genetic Medicine, School of Basic Medical Sciences, Fudan University, 200032 Shanghai, China; 2 Department of Otolaryngology, Shenzhen Key Laboratory of Nanozymes and Translational Cancer Research, Shenzhen Institute of Translational Medicine, The First Affiliated Hospital of Shenzhen University, Shenzhen Second People’s Hospital, 518035 Shenzhen, Guangdong China; 3 Department of Radiation Oncology, Jiangsu Cancer Hospital & Jiangsu Institute of Cancer Research & The Affiliated Cancer Hospital of Nanjing Medical University, Nanjing, Jiangsu China; 4 Department of Microbiology, Tumor and Cell Biology, Karolinska Institutet, Stockholm, Sweden; 5 Oujiang Laboratory (Zhejiang Lab for Regenerative Medicine, Vison and Brain Health), School of Pharmaceutical Science, Wenzhou Medical University, Wenzhou, P. R. China; 6 Longyan First Hospital Affiliated to Fujian Medical University, 364000 Longyan, Fujian China; 7 Department of Pathology, Shanghai Pulmonary Hospital, Tongji University School of Medicine, Shanghai, P. R. China; 8 Institute of Translational Medicine, Shanghai University, 99 Shangda Road, 200444 Shanghai, China; 9 Department of Oral Implantology, Stomatological Hospital and Dental School of Tongji University, Shanghai Engineering Research Center of Tooth Restoration and Regeneration, Shanghai, China

**Keywords:** Tumour angiogenesis, Head and neck cancer, Targeted therapies

## Abstract

Nasopharyngeal carcinoma (NPC) clinical trials show that antiangiogenic drugs (AADs) fail to achieve the expected efficacy, and combining AAD with chemoradiotherapy does not show superiority over chemoradiotherapy alone. Accumulating evidence suggests the intrinsic AAD resistance in NPC patients with poorly understood molecular mechanisms. Here, we describe NPC-specific FGF-2 expression-triggered, VEGF-independent angiogenesis as a mechanism of AAD resistance. Angiogenic factors screening between AAD-sensitive cancer type and AAD-resistant NPC showed high FGF-2 expression in NPC in both xenograft models and clinical samples. Mechanistically, the FGF-2-FGFR1-MYC axis drove endothelial cell survival and proliferation as an alternative to VEGF-VEGFR2-MYC signaling. Genetic knockdown of FGF-2 in NPC tumor cells reduced tumor angiogenesis, enhanced AAD sensitivity, and reduced pulmonary metastasis. Moreover, lenvatinib, an FDA recently approved multi-kinase inhibitor targeting both VEGFR2 and FGFR1, effectively inhibits the tumor vasculature, and exhibited robust anti-tumor effects in NPC-bearing nude mice and humanized mice compared with an agent equivalent to bevacizumab. These findings provide mechanistic insights on FGF-2 signaling in the modulation of VEGF pathway activation in the NPC microenvironment and propose an effective NPC-targeted therapy by using a clinically available drug.

## Introduction

Antiangiogenic drugs (AADs) are routinely used in patients with various types of solid cancer and effectively prolong patient survival. However, the responsiveness to AADs differs among tumor types. Clinically, bevacizumab, a recombinant humanized monoclonal antibody that neutralizes vascular endothelial growth factor-A (VEGF-A, or VEGF), is used in the first-line treatment of metastatic colorectal cancer (CRC) and produced significant survival improvement [1]. However, in certain types of tumors, AADs only produce limited therapeutic benefits or fail to provide any benefits. In 2011, FDA withdrew metastatic breast cancer from the bevacizumab indication list. Furthermore, most patients with pancreatic ductal adenocarcinoma show intrinsic resistance to bevacizumab [2]. Similar to patients with breast cancer or pancreatic cancer, adding bevacizumab to standard chemoradiotherapy did not show obvious superiority in patients with nasopharyngeal carcinoma (NPC) [3]. These clinical results demonstrate the AAD resistance in certain types of cancer, which is one of the major obstacles to current antiangiogenic therapy. In addition, a current impediment to the clinical use of AAD is the lack of reliable biomarkers to predict AAD therapeutic efficacy [4]. Although under intensive study [5], such biomarkers are still not clinically available.

Accounting for 73,000 deaths in 2018, NPC is an epithelial carcinoma with a specific geographical global distribution, with a high prevalence mainly in Southeast Asia [6, 7]. For early-stage NPC and non-metastatic NPC patients, chemoradiotherapy has shown satisfactory efficacy [8]. However, therapeutic options are still limited for metastatic NPC. Although AAD was recognized as an attractive approach to treating patients with NPC, the clinical results with bevacizumab resistance do not support this view [3]. To solve the bevacizumab resistance issue in NPC patients, in-depth mechanistic studies are urgently warranted. Commonly recognized mechanisms of AAD resistance include: (1) angiogenesis triggered by non-targeted growth factors [9]; (2) recruitment or activation of pro-angiogenic host cells [10, 11]; (3) vessel co-option or vessel remodeling [12, 13]; (4) endothelial cell (EC) transition [14]; and (5) metabolic shifts of tumor cells [15]. Of note, AAD resistance mechanisms, which have been thoroughly investigated in other tumor types, have not been reported in NPC.

The fibroblast growth factor (FGF) family consists of 18 members, which signal through the FGF receptor (FGFR) 1–4 on the membrane of various cell types [16]. Under physiological conditions, probably due to the compensation among the angiogenic factors, *Fgf2*^−/−^ *Fgf1*^−/−^ double knockout mice retain normal vascularization [17]. However, for tumor angiogenesis, it is clear that the FGF signaling regulates angiogenesis through RAS/MAPK, PI3K/Akt, Src tyrosine kinase, and STAT pathways [18]. In addition to VEGF, FGF-2 directly affects ECs through FGF receptors (FGFRs) to stimulate tumor neovascularization and vascular remodeling [19, 20]. Various FGFR inhibitors are currently under investigation in clinical trials [21]. Among them, lenvatinib, the only FDA-approved drug for solid tumors targeting both FGFR and VEGFR2, is used for treating advanced thyroid cancer and advanced hepatocellular carcinoma in clinical practice [22, 23]. Currently, little is known about the effects of lenvatinib on bevacizumab-resistant tumors.

In this study, we investigated the intrinsic AAD resistance mechanisms in NPC and found that NPC is an FGF-2 highly expressing tumor type. Gain- and loss-of-function experiments provide compelling evidence that FGF-2 compromises AAD efficacy through activation of compensatory FGF-2-FGFR1-MAPK-MYC signaling. By applying lenvatinib which targets both FGFR1 and VEGFR2 in NPC-bearing nude mice and humanized mice, we effectively overcome the AAD resistance in NPC. These findings provide a concept and rationale for improving the therapeutic efficacy of AADs in NPC patients, and for expanding the indications of FDA-approved lenvatinib.

## Results

### Intrinsic AAD resistance in NPC xenografts

**Fig. 1:**
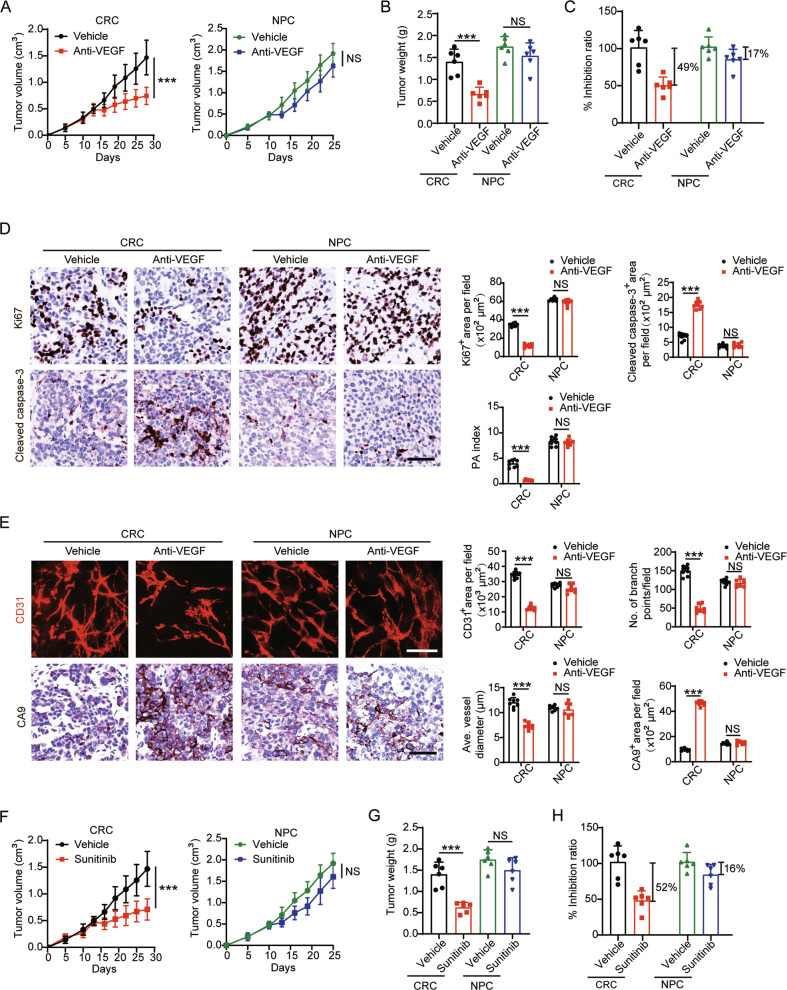
Tumor and vasculature responses to AAD in CRC and NPC. **A**–**C** Tumor growth (**A**) and tumor weights (**B**) were measured in CRC and NPC xenografts receiving vehicle or anti-VEGF treatment. The tumor inhibition ratios were calculated (**C**) (*n* = 6 mice per group). **D** Representative micrographs of Ki67^+^ proliferative cells and cleaved caspase-3^+^ apoptotic cells in vehicle- or anti-VEGF-treated CRC and NPC tumors. Scale bar = 50 μm. Quantification of Ki67^+^, cleaved caspase-3^+^ signals, and proliferation/apoptosis index (PA index) in vehicle- or anti-VEGF-treated CRC and NPC cancers (*n* = 8 random fields per group). **E** Representative micrographs of CD31^+^ microvessels and CA9^+^ hypoxic areas in vehicle- or anti-VEGF-treated CRC and NPC tumors. Scale bar in upper panel = 100 μm, scale bar in lower panel = 50 μm. Quantification of CD31^+^ tumor vessel parameters and CA9^+^ signals in vehicle- or anti-VEGF-treated CRC and NPC tumors (*n* = 8 random fields per group). **F**–**H** Tumor growth (**F**) and tumor weights (**G**) were measured in CRC and NPC xenografts receiving vehicle or sunitinib treatment. The tumor inhibition ratios were calculated (**H**) (*n* = 6 mice per group). ***P < 0.001. NS not significant. Data presented as mean ± SD.

In clinical practice, AADs have achieved great therapeutic effects in patients with CRC, and are used as first-line therapy for the treatment of metastatic CRC. However, similar effects are not observed in certain other types of cancers, such as NPC. To investigate whether NPC is an AAD-resistant tumor type, we established 5–8F human NPC and SW480 human CRC xenografts. NPC-bearing and CRC-bearing male nude mice were randomized into two groups and treated with an anti-VEGF neutralizing antibody (equivalent to bevacizumab) at 2.5 mg/kg or vehicle twice per week for two weeks. As expected in CRC-bearing mice, 2-week systemic treatment with VEGF blockade significantly inhibited tumor growth (Fig. 1A). Vehicle-treated CRCs grew to an average weight of 1.5 g at the endpoint (Fig. 1B). Anti-VEGF inhibited tumor growth by half in CRC xenografts (Fig. 1C). In contrast, in NPC-bearing mice, the same treatment did not reduce tumor growth or tumor weight, and the growth inhibition was only 17% (Fig. 1A–C). These interesting findings confirmed that NPC tumors are resistant to anti-VEGF-neutralizing antibodies.

AADs might inhibit tumor growth via alteration of the tumor microenvironment (TME). To investigate the TME changes in AAD-sensitive and AAD-resistant tumors, histological and immunofluorescence analyses were performed in CRC and NPC tumors. Indeed, VEGF blockade led to significant decreases of the Ki67^+^ proliferating tumor cell population in CRC, whereas cellular apoptosis in these treated tumors was markedly increased (Fig. 1D). Consequently, the proliferation/apoptosis (PA) index showed a drift toward an apoptotic phenotype (Fig. 1D). This phenotype supports our notion that CRC is AAD-sensitive. In contrast, anti-VEGF failed to induce an apoptotic phenotype in NPC, with insignificant effects on cell proliferation and apoptosis (Fig. 1D). To investigate whether AAD resistance in NPC depends on the tumor vasculature, we performed whole-mount immunofluorescence analyses for tumor microvessel visualization in both tumors. CRC tumors exhibited a marked reduction of microvessel density in the VEGF blockade-treated group (Fig. 1E). In addition, vascular branch points and vessel diameters significantly decreased in the anti-VEGF–treated group compared with those in the vehicle-treated group (Fig. 1E). Conversely, NPC tumors were resistant to anti-VEGF treatment, and kept their tumor vasculature characteristics upon treatment (Fig. 1E). Consistent with these findings, VEGF blockade treatments significantly increased tumor hypoxia in CRC but not in NPC (Fig. 1E). Improvement of tumor hypoxia in NPC might lead to increased tumor cell proliferation as measured by Ki67 positivity.

To further validate these findings, we used a clinically available TKI that targeted VEGFRs, sunitinib, for treating CRC and NPC tumor-bearing nude mice. Compared to same control groups, orally administrating 50 mg/kg of sunitinib every day for 2 weeks in xenograft models reproduced the results from the anti-VEGF neutralizing antibody experiment (Fig. 1F–H and Supplementary Fig. S1A, B). Together, these results demonstrate the intrinsic AAD resistance in NPC xenografts.

### NPC-specific FGF-2 expression in preclinical and clinical samples

**Fig. 2:**
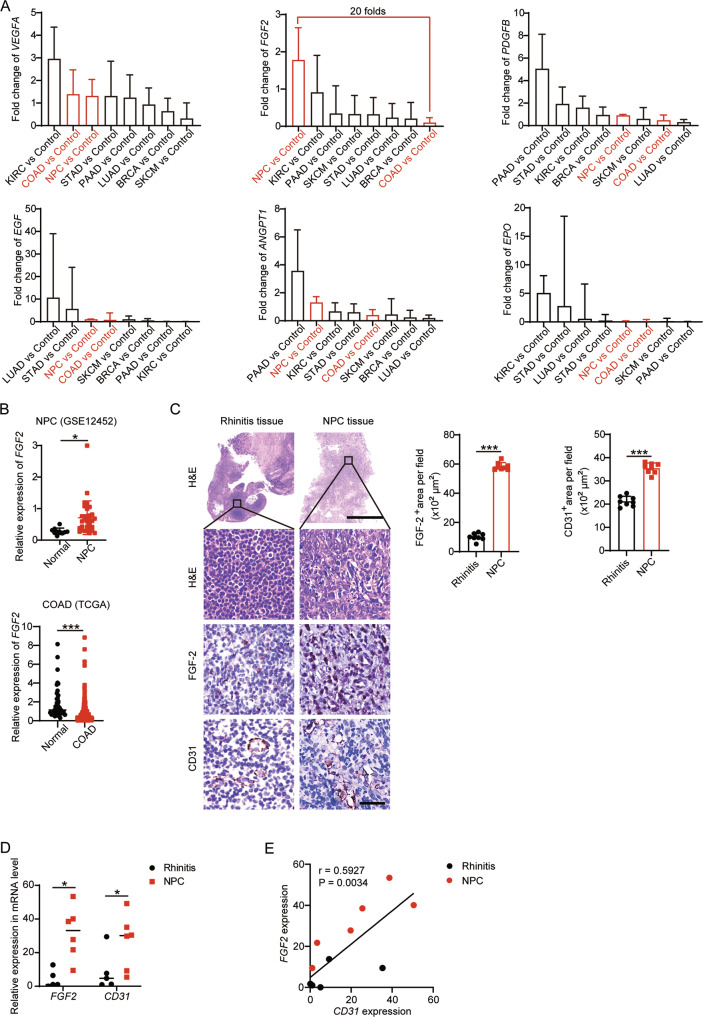
FGF-2 expression in NPC correlates with tumor vasculature. **A** Transcriptomic expression levels of angiogenic factors, including *VEGFA*, *FGF2*, *PDGFB*, *EGF*, *ANGPT1*, *EPO* in human KIRC tissues, COAD tissues, NPC tissues, STAD tissues, PAAD tissues, LUAD tissues, BRCA tissues, SKCM tissues, and their adjacent healthy tissues. The red line indicates the highest expression of *FGF2* in AAD-resistant NPC and the lowest expression of FGF in AAD-sensitive CRC. **B** Transcriptomic expression levels of *FGF2* in NPC tissues, COAD tissues, and their adjacent healthy tissues (sample number: control-NPC/NPC/control-COAD/COAD = 10/31/41/290). Data were extracted from datasets GSE12452 and TCGA. **C** Human rhinitis tissues and NPC tissues were collected and detected for histology (H&E), FGF-2, and CD31 expression levels. Scale bar in upper panel = 500 μm, scale bar in lower three panels = 50 μm. Quantification of FGF-2^+^ or CD31^+^ signals (*n* = 8 random fields per group). **D** QPCR quantification of *FGF2* and *CD31* expression in freshly collected rhinitis tissues and NPC tissues (rhinitis tissue, *n* = 5 samples; NPC tissue, *n* = 6 samples). **E** Correlation of *FGF2* and *CD31* transcriptomic expression levels of human NPCs and control rhinitis tissues (Rhinitis tissue, *n* = 5 samples; NPC tissue, *n* = 6 samples). *P < 0.05; ***P < 0.001. NS not significant. Data presented as mean ± SD. KIRC kidney renal clear cell carcinoma, COAD colon adenocarcinoma, STAD stomach adenocarcinoma, PAAD pancreatic adenocarcinoma, LUAD lung adenocarcinoma, BRCA breast invasive carcinoma, SKCM skin cutaneous melanoma.

Tumor cells might express non-VEGF angiogenic factors to compensate for VEGF signaling inhibition and sustain the tumor vasculature. To test whether NPC expresses high levels of non-VEGF angiogenic factors, we applied gene expression profiling to screen for the angiogenic factors expressed by NPC, CRC, and various other cancer types. Tissue RNA expression datasets were collected from The Cancer Genome Atlas (TCGA) and the Gene Expression Omnibus. Major cancer types include kidney renal clear cell carcinoma (KIRC), colon adenocarcinoma (COAD), NPC, stomach adenocarcinoma (STAD), pancreatic adenocarcinoma (PAAD), lung adenocarcinoma (LUAD), breast invasive carcinoma (BRCA), skin cutaneous melanoma (SKCM), and their corresponding controls were analyzed. We compared a panel of selected angiogenic factor genes, including VEGFs, FGFs, PDGF, EGF, EPO, ANGPT, and TPO, in these tumors and their adjacent control tissues. Interestingly, VEGF in AAD-resistant NPCs showed similar expression levels compared with AAD-sensitive CRCs (Fig. 2A). NPC and CRC expressed similar levels of these angiogenic factors except for FGF-2 (Fig. 2A and Supplementary Fig. S2A). Surprisingly, *FGF2* was exclusively highly expressed in AAD-resistant NPCs, but barely expressed in AAD-sensitive CRCs (Fig. 2A), resulting in a more than 20-fold difference. These findings were confirmed by independent analysis of tumor tissues and their corresponding healthy control tissues (Fig. 2B).

To validate our findings in clinical specimens, we analyzed previously collected paraffin sections of ten rhinitis tissues and six NPC tissues from patients receiving nasopharyngoscopy. Histochemistry staining of FGF-2 revealed a noteworthy FGF-2 expression in NPC tissues (Fig. 2C). Interestingly, compared with rhinitis control tissues, a markedly higher vessel density was observed in NPC. Quantification of CD31^+^ vascular density in NPC was significantly higher than that in rhinitis controls (Fig. 2C). To further investigate the correlation between FGF-2 and vasculature density, we collected fresh tissues of five rhinitis samples and six NPC samples and detected mRNA levels of *FGF2* and *CD31*. These two genes were both highly expressed in NPC tissues compared with rhinitis tissues (Fig. 2D). Correlation analysis revealed a strong correlation between *FGF2* and *CD31* expression, suggesting FGF-2-induced tumor angiogenesis (Fig. 2E). These data show that NPC-specific FGF-2 correlates with CD31 expression in the NPC microenvironment in patient samples.

### FGF-2 drives angiogenesis and metastasis in NPC-bearing mice

**Fig. 3:**
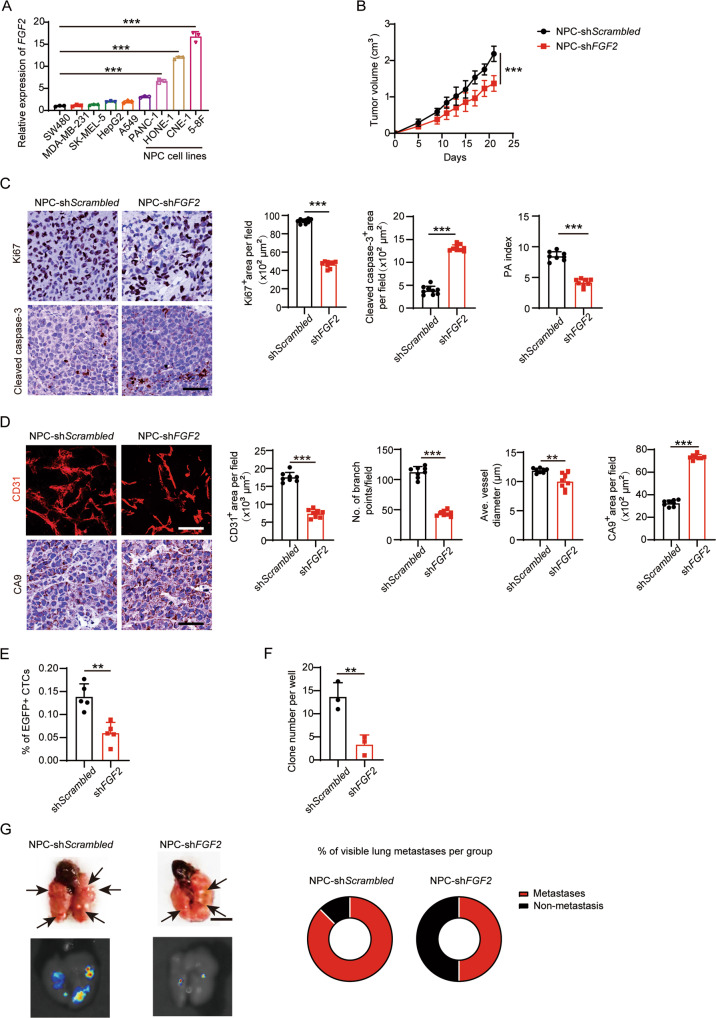
Tumor cell-derived FGF-2 promotes angiogenesis and metastasis in NPC xenograft models. **A** *FGF2* expression levels in SW480 CRC, MDA-MB-231 breast cancer, SK-MEL-5 melanoma, HepG2 hepatocellular carcinoma, A549 lung cancer, PANC-1 pancreatic ductal adenocarcinoma, HONE-1 NPC, CNE-1 NPC, and 5–8F NPC cell lines (*n* = 3 samples per group). **B** Tumor growth of scrambled or *FGF2* shRNA-transfected NPC tumor (*n* = 8 in NPC-sh*Scrambled* group and 6 in NPC-*FGF2* group). **C** Representative micrographs of Ki67^+^ proliferative cells and cleaved caspase-3^+^ apoptotic cells in NPC. Scale bar = 50 μm. Quantification of Ki67^+^, cleaved caspase-3^+^ signals, and PA index in NPC (*n* = 8 or 9 random fields per group). **D** Representative micrographs of CD31^+^ microvessels and CA9^+^ hypoxic areas in NPC. Scale bar in upper panel = 100 μm, scale bar in lower panel = 50 μm. Quantification of CD31^+^ tumor vessel parameters and CA9^+^ signals in NPC (*n* = 8 random fields per group). **E** Blood samples from tumor-bearing mice were FACS analyzed for EGFP^+^ signals. Quantification of EGFP^+^ circulating tumor cells (*n* = 5 samples per group). **F** Quantification of clones after culturing NPC-bearing mice blood samples for two weeks (*n* = 3 samples per group). **G** Representative graphs of lungs from NPC-bearing mice. Arrows indicate visible metastatic nodules. Scale bar = 0.5 cm. EGFP^+^ metastatic signals in the lung. Quantification of pulmonary metastasis proportion (*n* = 6 mice per group). **P < 0.01; ***P < 0.001. NS not significant. Data presented as mean ± SD.

To test the source of FGF-2 expression in NPC TME, *FGF2* mRNA levels were investigated in various human tumor cell lines. In concordance with clinical data, the CRC cell line showed the lowest *FGF2* expression, while three NPC cell lines, 5–8F, CNE-1, and HONE-1, expressed dramatically high levels of *FGF2* (Fig. 3A). We next chose the naturally high FGF-2-expressing human NPC 5–8F cells and performed *FGF2*-specific short hairpin RNA (shRNA)-knockdown experiments. Knockdown of *FGF2* markedly reduced NPC tumor growth following implantation in nude mice (Fig. 3B). PA index showed a drift toward an apoptotic phenotype compared to *FGF2*-competent controls (Fig. 3C). Interestingly, *FGF2* shRNA-transfected NPC significantly reduced microvessels, leading to an increase of tissue hypoxia (Fig. 3D). These results suggest FGF-2 promotes NPC angiogenesis. Tumor angiogenesis is closely correlated with metastasis. To further investigate the role of FGF-2 in NPC metastasis, we performed a series of metastasis-related experiments. As a result, knockdown of *FGF2* markedly suppressed circulating tumor cells (CTCs) by FACS detection, reduced tumor clone numbers in blood culture, and inhibited pulmonary metastasis (Fig. 3E–G). Pulmonary metastases were validated using gross examination and ex vivo visualization (Fig. 3G). These results suggested that FGF-2 promotes NPC metastasis in the xenograft model.

To validate FGF-2’s role in non-NPC tumors, we chose T241 mouse fibrosarcoma cells and 4T1 mouse breast cancer cells and genetically propagated them to stably express human FGF-2 [24]. Overexpressing FGF-2 facilitated tumor growth, proliferation phenotypes, angiogenesis, and metastasis in these mouse tumor models (Supplementary Fig. S3A–J), further supporting its angiogenic role and its functional consequences in the TME. Together, these results reveal upregulated tumor angiogenesis and metastasis in NPC and FGF-2-rich tumors.

### FGF-2-FGFR1-MYC signaling compensates for VEGF signaling in ECs

**Fig. 4:**
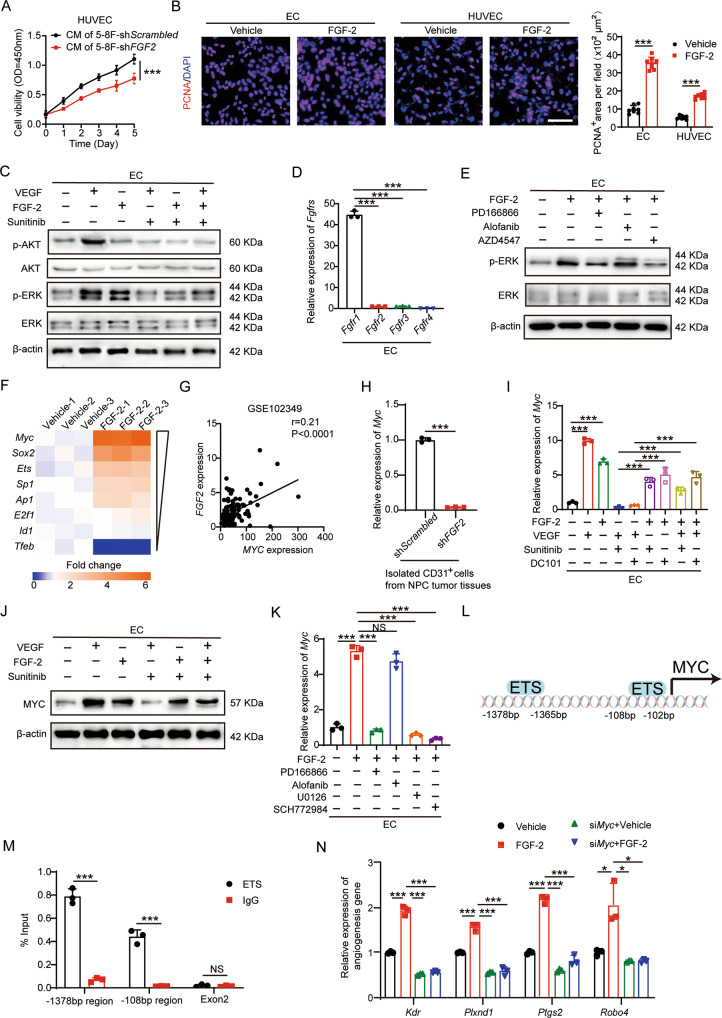
FGF-2 impedes the AAD-induced anti-EC effect via FGFR1-ERK-MYC signaling. **A** Cell growth of human ECs receiving the conditioned medium of scrambled- or *FGF2* shRNA-transfected NPC tumor cells (*n* = 5 samples per group). **B** Representative micrographs of PCNA^+^ proliferative cells and DAPI signals in ECs treated with vehicle or recombinant human FGF-2. Scale bar = 50 μm. Quantification of PCNA^+^ signals in mouse and human ECs (*n* = 8 random fields per group). **C** Vehicle- or VEGF-treated ECs were challenged with or without sunitinib or FGF-2. Phosphorylation of AKT and ERK in ECs was detected. β-actin marks the loading level in each lane (*n* = 3 samples per group). **D** QPCR quantification of *Fgfr1*, *Fgfr2*, *Fgfr3*, and *Fgfr4* mRNA levels in ECs (*n* = 3 samples per group). **E** Vehicle- or FGF-2-treated ECs were challenged with or without various FGFR inhibitors. Phosphorylation of ERK in ECs was detected. β-actin marks the loading level in each lane (*n* = 3 samples per group). **F** Downstream of VEGF signaling transcription factors were selected and detected in vehicle- or FGF-2-treated ECs. Heatmap of qPCR array screened out *Myc* as the highest upregulated transcription factor. **G** Correlation of *FGF2* and *MYC* transcriptomic expression levels of human NPCs (NPC, *n* = 113 samples). Data was extracted from dataset GSE102349. **H** QPCR quantification of *Myc* mRNA levels in isolated mouse CD31^+^ ECs from scrambled- or *FGF2* shRNA-transfected NPC tumor tissues (*n* = 3 samples per group). **I** QPCR quantification of *Myc* mRNA levels in various groups of ECs (*n* = 3 samples per group). **J** Vehicle- or VEGF-treated ECs were treated with or without AAD or FGF-2. MYC expression in ECs was detected. β-actin marks the loading level in each lane (*n* = 3 samples per group). **K** QPCR quantification of *Myc* mRNA levels in vehicle- or FGF-2-treated ECs, with or without various inhibitors (*n* = 3 samples per group). **L** Diagram of ETS-binding site prediction. **M** ChIP detection of ETS binding to the *Myc* gene promoter. Nonimmune IgG and *Myc* exon 2 regions served as controls (*n* = 3 samples per group). **N** QPCR quantification of EC proliferative marker *Kdr*, *Plxnd1*, *Ptgs2*, *Robo4* in scrambled- or *Myc* siRNA-transfected ECs administrated with vehicle or FGF-2 (*n* = 3 samples per group). *P < 0.05; **P < 0.01; ***P < 0.001. NS not significant. Data presented as mean ± SD.

Given the correlation between FGF-2 levels and angiogenesis in NPC tumor tissues, we investigated the functional consequence of FGF-2 on EC cells. The conditioned medium of NPC tumor cells increased EC proliferation, while knockdown of *FGF2* reduced this effect (Fig. 4A). In both human and mouse ECs, FGF-2 stimulated EC proliferation (Fig. 4B). These results are consistent with previously published data [20, 25]. Further, the multi-target kinase inhibitor sunitinib blocked VEGF-induced EC proliferation, while FGF-2 partially rescued this effect (Supplementary Fig. S4A). To investigate the detailed molecular mechanism, a VEGFR2-specific neutralizing antibody DC101 was used to inhibit VEGF-induced EC proliferation. Again, FGF-2 rescued EC proliferation following VEGFR2-dependent inhibition (Supplementary Fig. S4B). These results support the angiogenic role of FGF-2 is independent from VEGF signaling.

Next, downstream mechanisms were investigated by challenging ECs with vehicle, VEGF, or FGF-2, with or without AADs. VEGF activated both AKT and ERK pathways in a VEGFR-dependent manner, while FGF-2 only activated the ERK pathway (Fig. 4C and Supplementary Fig. S4C). Interestingly, both human and mouse ECs lacked FGFR2, R3, and R4 expression (Fig. 4D and Supplementary Fig. S4D). The FGF-2-FGFR1-ERK pathway was confirmed by treating ECs with the FGFR1-specific inhibitor PD166866, FGFR2-specific inhibitor alofanib, and FGFR pan-inhibitor AZD4547 (Fig. 4E). For mechanistic studies on the transcription level, we performed a literature study and listed the VEGF-regulated transcription factors including *Myc*, *Sox2*, *Ets*, *Sp1*, *Ap1*, *E2f1*, *Id1*, and *Tfeb*. A qPCR array analysis of FGF-2-stimulated ECs revealed that, among the VEGF-instigated transcription factors, MYC was the highest upregulated transcription factor upon FGF-2 challenge (Fig. 4F). Consistently, in NPC clinical datasets, *FGF2* was correlated with *MYC* expression (Fig. 4G). In isolated CD31^+^ ECs from control and sh*FGF2* NPC tumor tissues, knockdown *FGF2* in NPC tumor cells significantly reduced *MYC* expression in tumor-associated ECs (Fig. 4H and Supplementary Fig. S4E). Further studies confirmed that both VEGF and FGF-2 stimulate MYC expression. VEGFR2 inhibition blocked VEGF-induced MYC expression, while the addition of FGF-2 rescued MYC expression (Fig. 4I, J and Supplementary Fig. S4F). These data suggest that FGF-2 may compensate for VEGF signaling pathway through upregulating MYC expression. By applying an FGFR1-specific inhibitor PD166866, an FGFR2-specific inhibitor alofanib, a MEK1/2 inhibitor U0126, and an ERK1/2 inhibitor SCH772984, we confirmed FGF-2 promoted MYC expression via FGFR1-ERK signaling, but not via FGFR2 (Fig. 4K). It is reported that ETS supports MYC expression in NIH-3T3 cells by directly binding to its promoter region [26]. We next tested whether this mechanism exists in ECs. We analyzed the mouse *Myc* promoter region and found two ETS-binding sites at −1378 bp and −108 bp (Fig. 4L). Compared with the coding sequence, chromatin immunoprecipitation (ChIP) analysis reveals that ETS binds to the *Myc* promoter in these regions (Fig. 4M). In addition, other than promoting MYC expression, we found that FGF-2 also compensated for VEGF-instigated MYC phosphorylation (Supplementary Fig. S4G). It is reported that ERK may phosphorylate MYC for stabilization [27]. Using specific inhibitors, we found that MYC phosphorylation is FGFR1- and ERK-dependent (Supplementary Fig. S4H). These results demonstrate that the FGF2-FGFR1-ERK axis promotes MYC expression and phosphorylation.

Furthermore, knockdown of *MYC* using our previously validated siRNA [28] significantly impaired FGF-2-induced angiogenic gene signatures (Fig. 4N and Supplementary Fig. S4I). Together, we provided compelling mechanistic evidence demonstrating that FGF-2-FGFR1 signaling compensates for the VEGF-VEGFR2 signaling through upregulating the shared transcription factor MYC in ECs.

### Silencing of FGF-2 improves the AAD sensitivity in NPC

**Fig. 5:**
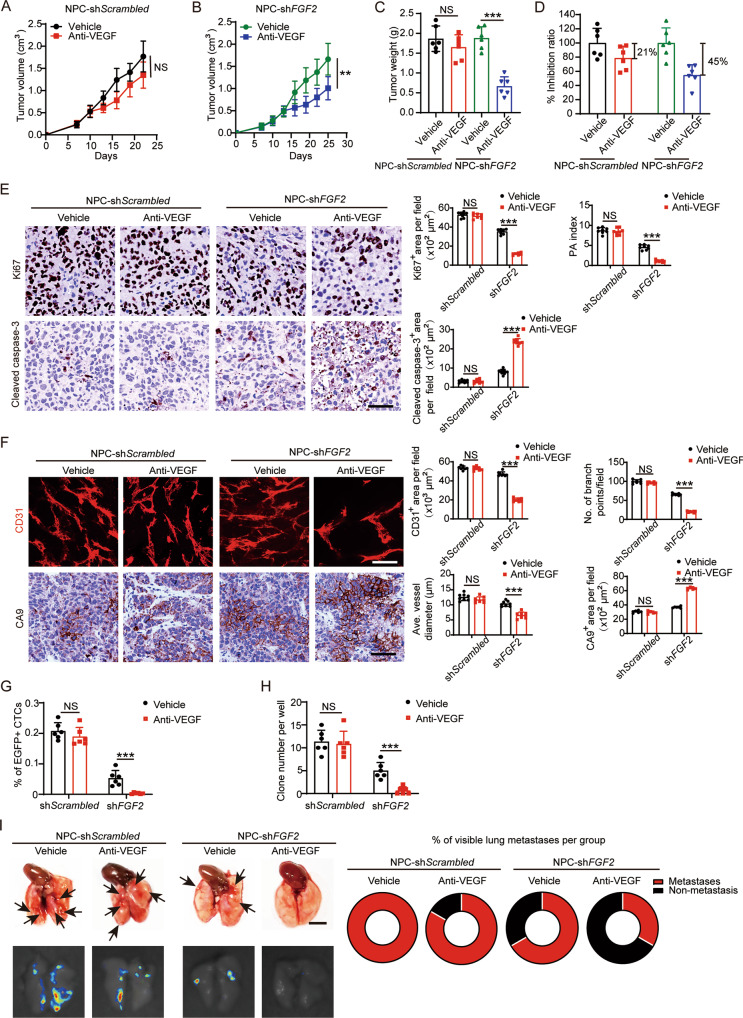
Knockdown of FGF2 in NPC tumor cells enhances AAD-mediated anti-tumor effects. **A**–**D** Tumor growth (**A,** **B**) and tumor weights (**C**) were measured in sh*Scrambled*- and sh*FGF2*-transfected NPC tumors receiving vehicle or anti-VEGF treatment. The tumor inhibition ratios were calculated (**D**) (*n* = 6 mice per group). **E** Representative micrographs of Ki67^+^ proliferative cells and cleaved caspase-3^+^ apoptotic cells in vehicle- or anti-VEGF-treated sh*Scrambled*- and sh*FGF2*-transfected NPC tumors. Scale bar = 50 μm. Quantification of Ki67^+^, cleaved caspase-3^+^ signals, and PA index in vehicle- or anti-VEGF-treated sh*Scrambled*- and sh*FGF2*-transfected NPC tumors (*n* = 8 random fields per group). **F** Representative micrographs of CD31^+^ microvessels and CA9^+^ hypoxic areas in vehicle- or anti-VEGF-treated sh*Scrambled*- and sh*FGF2*-transfected NPC tumors. Scale bar in upper panel = 100 μm, scale bar in lower panel = 50 μm. Quantification of CD31^+^ tumor vessel parameters and CA9^+^ signals in vehicle- or anti-VEGF-treated sh*Scrambled*- and sh*FGF2*-transfected NPC tumors (*n* = 8 random fields per group). **G** Blood samples from tumor-bearing mice were FACS analyzed for EGFP^+^ signals. Quantification of EGFP^+^ circulating tumor cells (*n* = 6 samples per group). **H** Quantification of clones after culturing blood samples from vehicle- or anti-VEGF-treated sh*Scrambled*- and sh*FGF2*-transfected NPC-bearing mice for two weeks (*n* = 6 samples per group). **I** Representative graphs of lungs from vehicle- or anti-VEGF-treated sh*Scrambled*- and sh*FGF2*-transfected NPC-bearing mice. Arrows indicate visible metastatic nodules. Scale bar = 0.5 cm. EGFP^+^ metastatic signals in the lung. Quantification of pulmonary metastasis proportion (*n* = 6 mice per group). **P < 0.01; ***P < 0.001. NS not significant. Data presented as mean ± SD.

To validate our findings in vivo, we treated NPC-sh*Scrambled* and NPC-sh*FGF2* tumor-xenografts with the anti-VEGF neutralizing antibody. As expected, knockdown of FGF-2 in NPC significantly increased the anti-tumor activity of the VEGF blockade, with the tumor inhibition rate improved to nearly 50% (Fig. 5A–D). The PA index showed that knockdown of FGF-2 decreased cell proliferation, tipping the balance toward an apoptotic phenotype (Fig. 5E). Markedly, NPC-sh*FGF2* tumors restored the antiangiogenic response to VEGF blockade (Fig. 5F). Consequently, CTCs and lung metastasis were significantly inhibited (Fig. 5G–I). These data suggest that the FGF-2 inhibition sensitizes otherwise resistant NPC tumors to the anti-VEGF drug, and further augments its tumor-suppressive effects.

### Lenvatinib is an effective therapy for treating AAD-resistant NPC

**Fig. 6:**
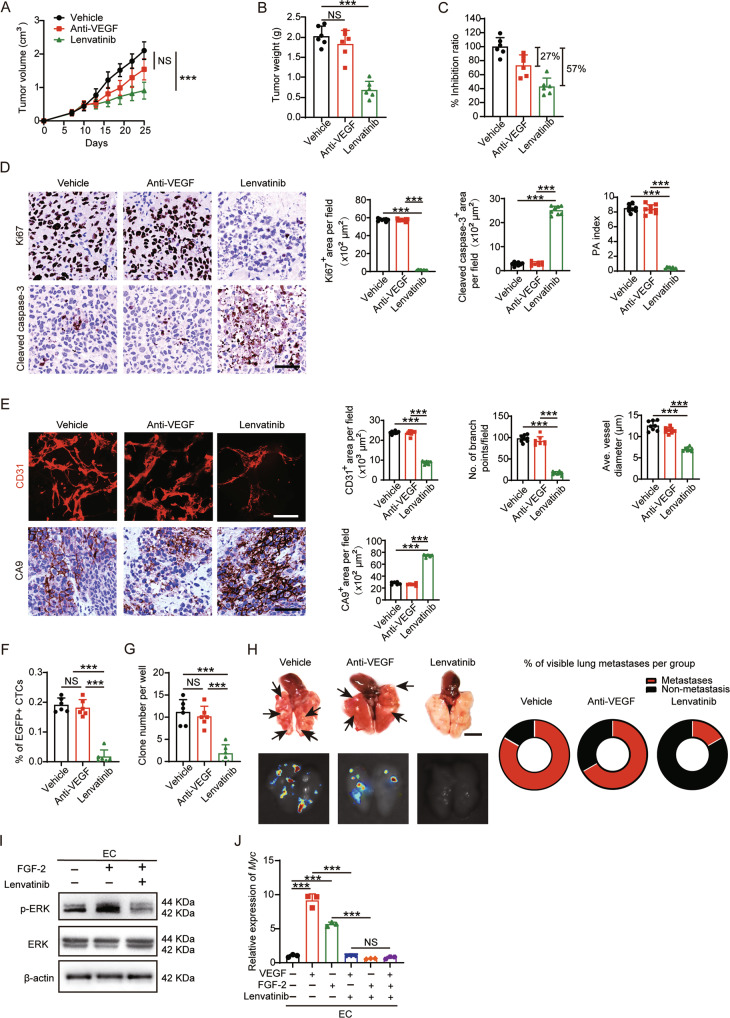
Lenvatinib effectively inhibits anti-VEGF-resistant NPC. **A**–**C** Tumor growth (**A**) and tumor weights (**B**) were measured in vehicle-, anti-VEGF-, and lenvatinib-treated NPC tumors. The tumor inhibition ratio were calculated (**C**) (*n* = 6 mice per group). **D** Representative micrographs of Ki67^+^ proliferative cells and cleaved caspase-3^+^ apoptotic cells in vehicle-, anti-VEGF-, and lenvatinib-treated NPC tumors. Scale bar = 50 μm. Quantification of Ki67^+^, cleaved caspase-3^+^ signals, and PA index in vehicle-, anti-VEGF-, and lenvatinib-treated NPC tumors. (*n* = 8 random fields per group) **E** Representative micrographs of CD31^+^ microvessels and CA9^+^ hypoxic areas in vehicle-, anti-VEGF-, and lenvatinib-treated NPC tumors. Scale bar in upper panel=100 μm, scale bar in lower panel=50 μm. Quantification of CD31^+^ tumor vessel parameters and CA9^+^ signals in vehicle-, anti-VEGF-, and lenvatinib-treated NPC tumors (*n* = 8 or 6 random fields per group). **F** Blood samples from tumor-bearing mice were FACS analyzed for EGFP^+^ signals. Quantification of EGFP^+^ circulating tumor cells (*n* = 6 samples per group). **G** Quantification of clones after culturing blood samples from vehicle-, anti-VEGF-, and lenvatinib-treated NPC-bearing mice for two weeks (*n* = 6 samples per group). **H** Representative graphs of lungs from vehicle-, anti-VEGF-, and lenvatinib-treated NPC-bearing mice. Arrows indicate visible metastatic nodules. Scale bar = 0.5 cm. EGFP^+^ metastatic signals in the lung. Quantification of pulmonary metastasis proportion (*n* = 6 mice per group). **I** Vehicle- or FGF-2-treated ECs were treated with or without lenvatinib. ERK phosphorylation in ECs was detected. β-actin marks the loading level in each lane (*n* = 3 samples per group). **J** QPCR quantification of *Myc* mRNA levels in vehicle- or FGF-2-treated ECs, with or without lenvatinib (*n* = 3 samples per group). ***P < 0.001. NS not significant. Data presented as mean ± SD.

Given the fact that FGF-2 signaling inhibition significantly promoted the therapeutic effects of AAD in NPC, we hypothesized that combination therapy targeting both FGF-2 and VEGF signaling may overcome the AAD resistance in NPC. We examined the clinically available drugs and found that, lenvatinib, used for treating hepatocellular carcinoma and thyroid cancer, is the only FDA-approved solid tumor drug targeting both FGFRs and VEGFRs. However, its efficacy in NPC has not been studied in preclinical or clinical settings. To test our hypothesis that lenvatinib overcomes AAD resistance in NPC, we treated NPC-bearing mice with anti-VEGF or lenvatinib at clinically relevant doses, both of which inhibited AAD-sensitive CRC for approximately 50% (Fig. 1 and Supplementary Fig. S5A). Surprisingly, lenvatinib significantly inhibited NPC growth, with a tumor inhibition rate of more than 50%. Anti-VEGF, as a negative control, exhibited an insignificant anti-tumor effect in NPC (Fig. 6A–C). The PA index showed that lenvatinib strongly induced an apoptotic phenotype (Fig. 6D) and inhibited the tumor vasculature (Fig. 6E), whereas VEGF blockade showed marginal effects (Fig. 6D and E). As a result, CTCs and lung metastasis were significantly inhibited in the lenvatinib but not in the anti-VEGF treatment group (Fig. 6F–H).

Mechanistically, to test whether lenvatinib successfully overcomes FGF-2-instigated angiogenesis, we examined the effect of lenvatinib on mouse ECs. As expected, similar to FGFR1 inhibitor (Fig. 4), lenvatinib significantly blocked ERK phosphorylation and MYC expression in ECs (Fig. 6I, J), supporting the proposed FGF2-FGFR1-ERK-MYC axis. Together, these data suggest that lenvatinib has anti-tumor potential to treat intrinsically AAD-resistant NPC.

### Lenvatinib suppresses NPC without altering the tumor immune microenvironment in NPC-bearing humanized mice

**Fig. 7:**
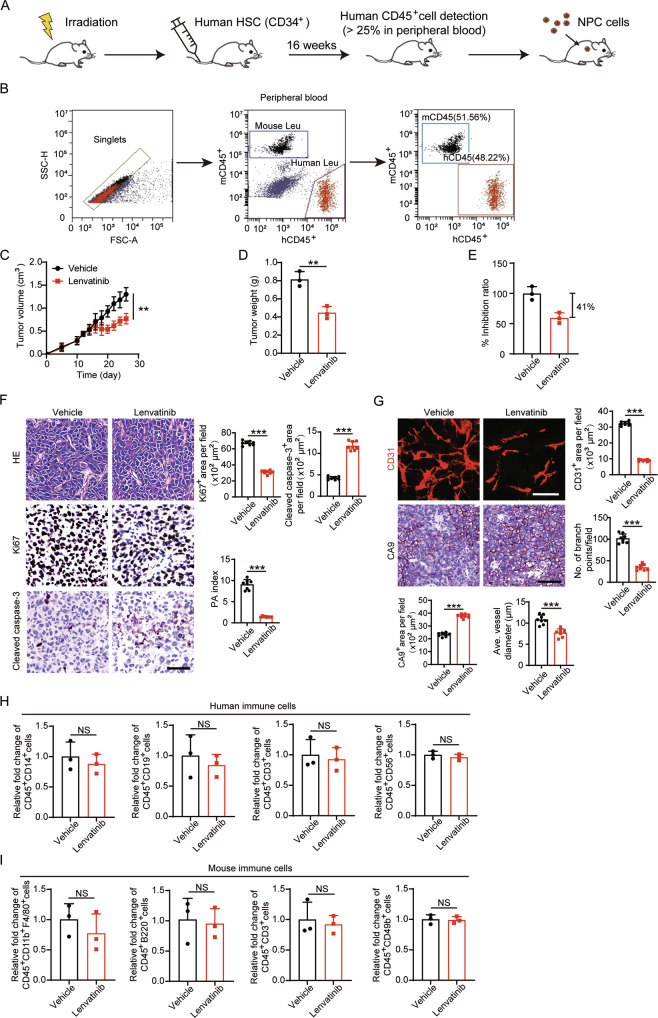
Lenvatinib insignificantly affects the immune microenvironment in NPC in humanized NSG mice. **A** Schematic diagram of the establishment of humanized NSG mice. **B** Representative FACS analysis of human CD45^+^ cells in mouse peripheral blood. Human CD45^+^ cell percentage greater than 25% was considered successful in modeling. **C**–**E** Tumor growth (**C**), tumor weights (**D**) were measured in vehicle-, anti-VEGF-, and lenvatinib-treated NPC tumors. The tumor inhibition ratio were calculated (**E**) (*n* = 3 samples per group). **F** Representative micrographs of Ki67^+^ proliferative cells and cleaved caspase-3^+^ apoptotic cells in vehicle-, anti-VEGF-, and lenvatinib-treated NPC tumors. Scale bar = 50 μm. Quantification of Ki67^+^, cleaved caspase-3^+^ signals, and PA index in vehicle-, anti-VEGF-, and lenvatinib-treated NPC tumors. (*n* = 8 random fields per group) **G** Representative micrographs of CD31^+^ microvessels and CA9^+^ hypoxic areas in vehicle-, anti-VEGF-, and lenvatinib-treated NPC tumors. Scale bar in upper panel = 100 μm, scale bar in lower panel = 50 μm. Quantification of CD31^+^ tumor vessel parameters and CA9^+^ signals in vehicle-, anti-VEGF-, and lenvatinib-treated NPC tumors (*n* = 8 random fields per group). **H** Quantification of hCD45^+^ hCD14^+^ population, hCD45^+^ hCD19^+^ population, hCD45^+^ hCD3^+^ population, and hCD45^+^ hCD56^+^ population in the NPC TME (*n* = 3 samples per group). **I** Quantification of mCD45^+^ mCD11b^+^ mF4/80^+^ population, mCD45^+^ mB220^+^ population, mCD45^+^ mCD3^+^ population, and mCD45^+^ mCD49b^+^ population in the NPC TME (*n* = 3 samples per group). **P < 0.01; ***P < 0.001. NS not significant. Data presented as mean ± SD.

Clinically, the tumor immune microenvironment (TIME) affects the efficacy of tumor therapy, and differences in the TIME between mouse models and humans may impede the clinical translation of a proposed therapy [29]. It has been reported that both FGF-2 and VEGF affect the tumor immune landscape [30, 31]. To test whether lenvatinib alters the TIME in a clinically relevant setting, we used humanized immune-deficient mice in which we examined the anti-angiogenesis capacity, anti-tumor capacity, and immune modulation capacity of lenvatinib. By transplanting human hematopoietic stem cells and progenitor cells into an irradiated NOD SCID Gamma (NSG) mouse strain (Fig. 7A), we successfully constructed humanized NSG mice with nearly 50% of CD45^+^ cells in the peripheral blood from the human origin (Fig. 7B). Human NPC cells were inoculated in this strain to establish the human NPC microenvironment. Of note, we found that tumor growth was relatively slower in humanized NSG mice compared to nude mice (Figs. 6A and 7C). By treating with equal amounts of lenvatinib, the tumor suppression rate in humanized NSG mice was still over 40%, compared with a 57% suppression in nude mice (Figs. 7C–E and 6C). Similarly, tumors in the lenvatinib-treated group showed a significant propensity for apoptosis (Fig. 7F), and the vascular inhibition rate was similar to that of the nude mouse model (Fig. 7G). These data suggest that the antiangiogenic properties and anti-tumor capacity of lenvatinib are not affected by the human immune cells. We further examined the proportion of immune cells in the TIME using flow cytometry analysis after tissue digestion (Supplementary Fig S6A, B) and found that human CD45^+^ CD14^+^ monocytes, CD45^+^ CD19^+^ B cells, CD45^+^ CD3^+^ T cells, and CD45^+^ CD56^+^ NK cells in NPC tumors were not affected by lenvatinib treatment (Fig. 7H). Similarly, mouse CD45^+^ CD11b^+^ F4/80^+^ macrophages, CD45^+^ B220^+^ B cells, CD45^+^ CD3^+^ T cells, and CD45^+^ CD49b^+^ NK cells remained unaltered (Fig. 7I). These data suggest that lenvatinib does not alter the TIME in NPC, which provides an opportunity to combine lenvatinib with immunotherapy for additional or synergetic tumor-suppressive effects in the future.

## Discussion

In AAD clinical practice, drug resistance is currently the biggest challenge for patients with solid tumors. Although various mechanisms of drug resistance have been proposed [32], the most commonly described mechanism is the compensatory mechanism, in which a non-VEGF angiogenic factor is produced as an alternative to drive tumor angiogenesis [33]. This compensatory mechanism can be further divided into intrinsic and extrinsic mechanisms. The former commonly occurs when tumor cells enhance non-VEGF angiogenic factor expression through signaling by oncogenes, while the latter can be mediated by drug-induced microenvironmental stress, including AAD-triggered tissue hypoxia. In either case, a common anti-AAD resistance strategy in preclinical studies is combination therapy targeting both VEGF signaling and non-VEGF angiogenic signaling [10, 15, 34]. However, the rationale and clinical implementation of combination therapy face many open questions: Are the mechanisms sufficiently clear? Are the pharmacokinetics clear? In what ratio and regimen should the drug be used? Are there any additional side effects? These open questions have hindered the FDA approval of previously proposed combination therapies [35]. In this regard, high hopes are instead placed on bifunctional molecules, which have predictable pharmacokinetic profiles, improved patient compliance, low cost, and no drug-drug interactions [36]. Ideally, the two targets of the drug should be non-redundant and not interfere with each other, and the physical combination of the two active domains into one drug should provide better targeting, hence less toxicity, compared with monotherapy. It would be an attractive strategy to overcome AAD resistance with a multi-targeted drug. Other than combination therapy or multi-targeted drug, resolution of AAD resistance may be achieved by various methods, such as increasing drug concentration, optimizing the therapeutic regimen, or precise drug delivery. Exploring these possibilities in the clinical setting is costly. It is warranted to further explore these possibilities using clinical-relevant animal models [37].

**Fig. 8:**
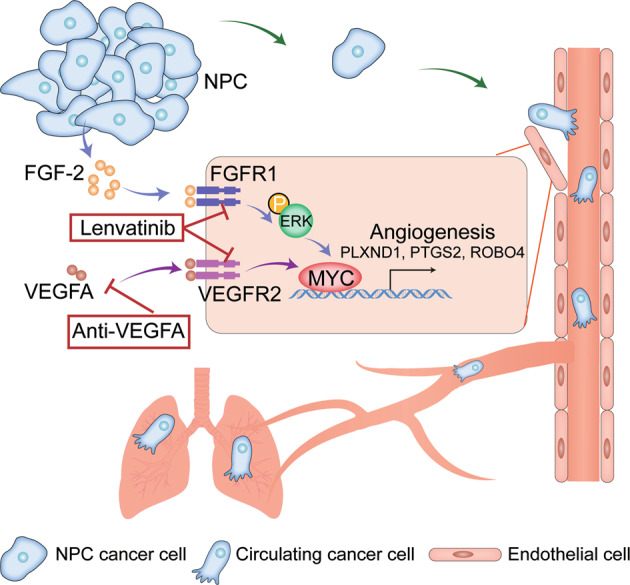
Schematic diagram of FGF-2-FGFR1-ERK-MYC axis mediates AAD resistance and metastasis in NPC. NPC cancer cells often produce FGF-2, and FGF-2 impedes the AAD-mediated anti-EC effect in the TME. In FGF-2 positive tumors, the FGFR1-ERK-MYC axis is upregulated in ECs and compensates for VEGF-VEGFR2-MYC signaling. MYC further drives downstream angiogenic genes, hence keep the angiogenesis in the TME. Angiogenic tumor vessels further facilitate tumor cell intravasation and pulmonary metastasis. In such FGF-2 high TME, inhibition of VEGF-VEGFR2 signaling alone does not effectively inhibit angiogenesis. Lenvatinib, an FDA-approved multi-kinase inhibitor targeting both VEGFR2 and FGFR1, exhibits robust antiangiogenic and anti-tumor effects in NPC or possibly FGF-2-rich tumors.

One of the surprising findings of our work is that, by comparing various angiogenic factors across multiple datasets and between AAD-sensitive and resistant tumors, we found that NPC specifically expressed high levels of the angiogenic factor, FGF-2. This feature of NPC has not been reported previously. This non-VEGF angiogenic factor leads to an intrinsic AAD resistance and explains the poor clinical efficacy of AAD in NPC patients. In the field, FGF-2-induced AAD resistance has been described in other cancers [13, 18]. However, the spatiotemporal expression of FGF-2 in the tumor microenvironment is unclear. We show that in NPC, it is synthesized by tumor cells and stimulates angiogenesis via FGFR1 on the ECs. Mechanistically, FGF-2 compensates for the lack of VEGF-VEGFR-MYC pathway activation by upregulating MYC in ECs. These works provide mechanistic insights into FGF-2-triggered AAD resistance in NPC (Fig. 8). Notably, in the current work, we only studied this compensatory effect in ECs for studying tumor angiogenesis, whether this mechanism exists in other cell components in the TME is yet to be investigated.

Lenvatinib is used as the first-line treatment of hepatocellular carcinoma and thyroid cancer [38]. There are a large number of clinical trials designed for expanding its indications either alone or in combination with immunotherapy, targeting biliary tract cancer, renal cell carcinoma, osteosarcoma, and malignant melanoma (clinicaltrials.gov). However, there are currently no ongoing trials or reported plans to use lenvatinib for NPC treatment. In our work, we exploited that lenvatinib targets FGFRs and proved the concept of overcoming AAD resistance by using lenvatinib for the treatment of FGF-2-expressing tumors. If our preclinical work can be translated to clinical therapy, targeting FGFR would offer an attractive approach to improving drug sensitivity and clinical benefits of AAD (Fig. 8). Perhaps, lenvatinib or combining lenvatinib with another drug may provide better clinical benefits than current therapeutic options for patients with NPC. This view deserves further clinical validation. Currently, in other cancer types, the combination of AAD with immune checkpoint inhibitors contributes to antitumor efficacy [39, 40]. Using humanized NSG mouse model, we provided compelling evidence that lenvatinib insignificantly affects the TIME of NPC, which provides an opportunity to combine lenvatinib with immune checkpoint inhibitors to obtain greater efficacy in clinical practice.

Moreover, based on our results, it is reasonable to speculate that detection of FGF-2 expression in NPC tumors may predict the therapeutic efficacy of lenvatinib. This point of view may extend to other FGF-2-expressing tumors.

Taken together, our findings show that FGF-2 is highly expressed in NPC xenografts and NPC clinical samples, and provide a mechanistic explanation of the intrinsic AAD resistance mediated through FGF-2-FGFR1-MAPK-MYC signaling. These findings provide a concept and rationale for improving the therapeutic efficacy of AADs in NPC patients, and pave potential avenues for treating NPC patients with FDA-approved lenvatinib.

## Materials and methods

### Cell culture

Human 5–8F, HONE-1, CNE-1 NPC cell lines were kindly provided by Prof. Zesong Li at the Shenzhen Second People’s Hospital, Shenzhen, China. Human A549 lung carcinoma cell line was kindly provided by Dr. Yongbo Wang at the Fudan University, Shanghai, China. Human SK-MEL-5 melanoma, HepG2 hepatocellular carcinoma, MDA-MB-231 breast cancer, SW480 colorectal cancer, PANC-1 pancreatic carcinoma, 293 T embryonic kidney cell line, murine T241 fibrosarcoma, murine 4T1 breast cancer, and isolated primary murine endothelial cells were kindly provided by Prof. Yihai Cao at the Karolinska Institute, Stockholm, Sweden. Human HUVEC endothelial cells were purchased from ATCC. T241 and 4T1 cells were transfected with human FGF-2 and control vector for FGF-2 overexpression. Sh*Scrambled* vector and sh*FGF2* vector were transfected into 5–8F cell lines with GFP using a lentiviral system (GeneCopoeia Inc., USA) for FGF-2 knockdown [24]. SW480, A549, HONE-1, CNE-1, and 5–8F cell lines were cultured in 10% FBS supplemented RPMI 1640 (Cat. No. 40130ES76, YEASEN, China; Cat. No. MA0215, Meilunbio, China), containing 100 U/mL penicillin, 100 μg/mL streptomycin (Cat. No. MA0110, Meilunbio, China). T241, 4T1, PANC-1, SK-MEL-5, HepG2, MDA-MB-231, and 293 T cell lines were cultured in 10% FBS supplemented DMEM (Cat. No. MA0213, Meilunbio, China) with penicillin/streptomycin. HUVEC and mouse endothelial cells were cultured in 10% FBS supplemented M199 (Cat. No. SH30253.01, HyClone) with penicillin/streptomycin. All cell lines used in our study were not authenticated using STR profiling and were tested negative for mycoplasma [24].

### Cell isolation

Cells were digested and isolated using a magnetic-activated cell sorting (MACS) method [41]. In brief, tissues were cut into small pieces and digested in 0.1% collagenase I and II in PBS (Cat. No. 40507ES60, YEASEN; Cat. No. 40508ES60, YEASEN; Cat. No. MA0015, Meilunbio, China) in 37 °C for 30 min with gentle pipetting. Cells were washed and resuspended by 1 mL MACS buffer (0.5% BSA, 2 mM EDTA in PBS), and stained with a goat anti-mouse CD31 antibody (Cat. No. AF3628, R&D), followed by an Alexa Fluor 647-conjugated donkey anti-goat antibody (Cat. No. A21447, Invitrogen). Magnetic labeling was performed with Anti-Alexa Fluor 647 MicroBeads (Cat. No. 130-091-395, Miltenyi Biotec; Cat. No. 130-042-303, Miltenyi Biotec). To sort CD31^+^ cells, a column and magnetic MACS separators (Cat. No. 130-042-201, Miltenyi Biotec) were used. Cells were collected for the following experiments.

### Animals

All animal studies were approved by the Animal Experimental Ethical Committee of the Fudan University, Shanghai, China (No. 20200306-071). Male C57BL/6, female BALB/c, and male BALB/c-nu/nu nude mice at the age between 6- and 8-week-old were purchased from GemPharmatech, China, and maintained under a 12-h dark/12-h light cycle with food and water provided ad libitum. Humanized NSG mice were constructed by Shanghai Model Organisms, China. In brief, human CD34^+^ cells were isolated from cord blood and were transplanted into each 1.4 Gy-irradiated NSG mouse. After 16 weeks, the humanization of each mouse is analyzed by detecting the percentage of human CD45^+^ cells in all CD45^+^ cells in peripheral blood. Human CD45^+^ cell percentage greater than 25% was considered successful in modeling. All animals were randomly assigned to groups before experiments. No statistical methods were used to predetermine the sample size. The experimenter was not blind to the assignment of the groups and the evaluation of the results. No samples, animals, or data were excluded.

### Human patient samples

All human studies were approved by the Ethical Review Committee in the Shenzhen Second People’s Hospital, Shenzhen, China (No. 20200525002). Fresh samples were collected from patients receiving nasopharyngoscopic biopsies. Informed consent was obtained from all subjects.

### Tumor model and metastasis model

For subcutaneous tumor models, approximately 1 × 10^6^ tumor cells in 100 μL PBS were subcutaneously implanted into each mouse. For T241, 4T1, and 5–8F were subcutaneously or orthotopically injected into C57BL/6, BALB/c, and BALB/c-nu/nu nude mice, respectively. Every other day, tumor volumes were measured and calculated according to a standard formula [15]. For metastasis detection, at a primary tumor volume of 2.0 cm^3^, the primary tumor was surgically removed under anesthesia. After surgery, mice were kept for an additional 4–6 weeks to detect metastases. An IVIS system (VISQUE Invivo Elite, Vieworks, Korea) was used to detect GFP^+^ nodules in the lung. All tumor experiments in this study did not exceed 2.5 cm^3^ tumor volume.

### Histological analysis, immunohistochemistry, immunofluorescence, and whole-mount

Histological analysis was performed using 4% paraformaldehyde (PFA) (Cat. No. MA0192, Meilunbio, China) fixed tissues. Paraffin-embedded tissues were cut into the thickness of 5 µm, mounted onto glass slides, baked for 1 h at 60 °C, deparaffinized in Xylene (Cat. No. 10023418, SCR, China), and sequentially rehydrated in 99%, 95%, and 70% ethanol (Cat. No. 10009218, SCR, China). Tissue slides were counterstained with Haematoxylin (Mayer’s) (Cat. No. MB9897, Meilunbio, China) and Eosin (Cat. No. MA0164, Meilunbio, China). Stained tissues were analyzed under a light microscope (Leica DM IL LED). Immunohistochemical staining was performed using paraffin-embedded tissues. Tissue slides were stained with a rabbit anti-Ki67 antibody (Cat. No. ab16667, Abcam, 1:200); a rabbit anti-Cleaved caspase-3 antibody (Cat. No. 9664, Cell Signaling Technology, 1:100); a rabbit anti-CA9 antibody (Cat. No. 11071-1-AP, Proteintech, 1:200), and a rabbit anti-FGF-2 antibody (Cat. No. A0235, ABclonal, 1:100). After rinsing, tissue samples were further stained by immunohistochemistry secondary antibodies, an anti-rabbit IgG (HRP) antibody (Cat. No. ab205718, Abcam). For immunofluorescence staining, cells seeded on glass coverslips were stained with a mouse anti-PCNA antibody (Cat. No. BM0104, BOSTER, 1:800). After rising, cell samples were further stained for 30 min with a donkey anti-mouse Alexa Fluor 594 antibody (Cat. No. A21203, Invitrogen, 1:400). Nuclei were counterstained with DAPI (Cat. No. MA0128, Meilunbio, China). Positive signals were captured using a fluorescence microscope (Olympus BX53, Japan). Whole-mount staining was performed using the standard method [42, 43]. Briefly, tumor tissues were cut into small pieces and digested with 20 mM proteinase K in 10 mM Tris buffer (pH 7.5) for 5 min, followed by incubation with 100% methanol for 30 min. Tissues were washed with PBS and incubated at 4 °C overnight in PBS containing 3% skim milk and 0.3% Triton X-100, followed by incubation of a goat anti-mouse CD31 monoclonal antibody (Cat. No. AF3628, R&D, 1:300). After rinsing, tissue samples were further stained for 2 h at room temperature with secondary antibody, a donkey anti-goat Alexa 555 antibody (Cat. No. A21432, Invitrogen, 1:200). After thorough washing, slides were mounted using the anti-fading mounting medium (Cat. No. MA0235, Meilunbio, China) and examined under a confocal microscope system (X-LIGHT V2 spinning disk confocal, 89 North; Leica DMi8 microscope, Leica). Captured images were further analyzed using an Adobe Photoshop CS software program.

### RNA extraction and quantitative real-time PCR

Total RNAs were extracted from various tissues and cultured cells using an RNA simple Total RNA kit (Cat. No. DP419, TIANGEN, China). Total RNA from each sample was reversely transcribed using a Hifair^®^ II 1st Strand cDNA Synthesis SuperMix (Cat. No. 11123ES60, YEASEN, China). Reverse transcription was performed at 42 °C for 30 min, subsequently 85 °C for 5 min to inactivate the enzyme activity. The cDNA samples were subjected to qPCR using a StepOnePlus Real-Time PCR System (Applied Biosystems). Each sample was triplicated and in a 10 μL reaction containing Hieff^®^ qPCR SYBR Green Master Mix (Cat. No. 11203ES03, YEASEN, China), 50 nM forward and reverse primers and 2 μL cDNA. The qPCR protocol was executed for 40 cycles and each cycle consisted of denaturation at 95 °C for 15 s, annealing at 60 °C for 1 min, and extension at 72 °C for 1 min. The primer pairs specific for various genes used in our experiments included: human *FGF2* forward: 5’-AGAAGAGCGACCCTCACATCA-3’; human *FGF2* reverse: 5’-CGGTTAGCACACACTCCTTTG-3’; human *CD31* forward: 5’-AACAGTGTTGACATGAAGAGCC-3’; human *CD31* reverse: 5’-TGTAAAACAGCACGTCATCCTT-3’; human *FGFR1* forward: 5’-CCCGTAGCTCCATATTGGACA-3’; human *FGFR1* reverse: 5’-TTTGCCATTTTTCAACCAGCG-3’; human *FGFR2* forward: 5’-AGCACCATACTGGACCAACAC-3’; human *FGFR2* reverse: 5’-GGCAGCGAAACTTGACAGTG-3’; human *FGFR3* forward: 5’-TGCGTCGTGGAGAACAAGTTT-3’; human *FGFR3* reverse: 5’-GCACGGTAACGTAGGGTGTG-3’; human *FGFR4* forward: 5’-GAGGGGCCGCCTAGAGATT-3’; human *FGFR4* reverse: 5’-CAGGACGATCATGGAGCCT-3’; human *MYC* forward: 5’-GGCTCCTGGCAAAAGGTCA-3’; human *MYC* reverse: 5’-CTGCGTAGTTGTGCTGATGT-3’; human *GAPDH* forward: 5’-CTGGGCTACACTGAGCACC-3’; human *GAPDH* reverse: 5’-AAGTGGTCGTTGAGGGCAATG-3’; mouse *Fgf2* forward: 5’-GCGACCCACACGTCAAACTA-3’; mouse *Fgf2* reverse: 5’-TCCCTTGATAGACACAACTCCTC-3’; mouse *Fgfr1* forward: 5’-TAATACCACCGACAAGGAAATGG-3’; mouse *Fgfr1* reverse: 5’-TGATGGGAGAGTCCGATAGAGT-3’; mouse *Fgfr2* forward: 5’-CCTCGATGTCGTTGAACGGTC-3’; mouse *Fgfr2* reverse: 5’-CAGCATCCATCTCCGTCACA-3’; mouse *Fgfr3* forward: 5’-GCCTGCGTGCTAGTGTTCT-3’; mouse *Fgfr3* reverse: 5’-TACCATCCTTAGCCCAGACCG-3’; mouse *Fgfr4* forward: 5’-GCTCGGAGGTAGAGGTCTTGT-3’; mouse *Fgfr4* reverse: 5’-CCACGCTGACTGGTAGGAA-3’; mouse *Myc* forward: 5’-ATGCCCCTCAACGTGAACTTC-3’; mouse *Myc* reverse: 5’-CGCAACATAGGATGGAGAGCA-3’; mouse *Sox2* forward: 5’-GCGGAGTGGAAACTTTTGTCC-3’; mouse *Sox2* reverse: 5’-CGGGAAGCGTGTACTTATCCTT-3’; mouse *Ets* forward: 5’-TCCTATCAGCTCGGAAGAACTC-3’; mouse *Ets* reverse: 5’-TCTTGCTTGATGGCAAAGTAGTC-3’; mouse *Sp1* forward: 5’-GCCGCCTTTTCTCAGACTC-3’; mouse *Sp1* reverse: 5’-TTGGGTGACTCAATTCTGCTG-3’; mouse *Ap1* forward: 5’-CCTTCTACGACGATGCCCTC-3’; mouse *Ap1* reverse: 5’-GGTTCAAGGTCATGCTCTGTTT-3’; mouse *E2f1* forward: 5’-CAGAACCTATGGCTAGGGAGT-3’; mouse *E2f1* reverse: 5’-GATCCAGCCTCCGTTTCACC-3’; mouse *Id1* forward: 5’-GGTCCGAGGCAGAGTATTACA-3’; mouse *Id1* reverse: 5’-CCTGAAAAGTAAGGAAGGGGGA-3’; mouse *Tfeb* forward: 5’- CCACCCCAGCCATCAACAC-3’; mouse *Tfeb* reverse: 5’-CAGACAGATACTCCCGAACCTT-3’; mouse *Kdr* forward: 5’- TTTGGCAAATACAACCCTTCAGA-3’; mouse *Kdr* reverse: 5’- GCAGAAGATACTGTCACCACC-3’; mouse *Plxnd1* forward: 5’- TCGCTGCCAATCCCTAATAAGA-3’; mouse *Plxnd1* reverse: 5’-TGACCTGGTTTGGAACTGTTG-3’; mouse *Ptgs2* forward: 5’- TTCAACACACTCTATCACTGGC-3’; mouse *Ptgs2* reverse: 5’-AGAAGCGTTTGCGGTACTCAT-3’; mouse *Robo4* forward: 5’- GCCTCCTTTTAGGTGAGGGAA-3’; mouse *Robo4* reverse: 5’-TGAGGGGGACCAACAGACAG-3’; mouse *Gapdh* forward: 5’- AGGTCGGTGTGAACGGATTTG-3’; mouse *Gapdh* reverse: 5’-TGTAGACCATGTAGTTGAGGTCA-3’.

### Immunoblot

Cells were lysed in a RIPA lysis buffer containing proteinase and phosphatase inhibitor cocktails (Cat. No. MA0151, Meilunbio, China; Cat. No. MB2678, Meilunbio, China; 1:100) for total protein collection. Protein samples and a standard molecular weight marker (Cat. No. AP13L052, Life-iLab, China) were loaded on a 10% SDS-PAGE gel (Cat. No. AP15L945, Life-iLab, China), and transferred onto a polyvinylidene difluoride (PVDF) membrane (Cat. No. IPVH00010, Millipore), which was subsequently blocked with 5% skimmed milk for 2 h. Membranes were incubated overnight at 4 °C with primary antibodies diluted in a Primary Antibody Dilution Buffer (Cat. No. MB9881, Meilunbio, China). After rigorous washing with PBS containing 0.1% Tween-20 (Cat. No. T8220, Solarbio, China), membranes were incubated at room temperature for 1 h with a goat anti-mouse HRP-conjugated IgG antibody (Cat. No. AS003, ABclonal, China; 1:5000) or a goat anti-rabbit HRP- conjugated IgG antibody (Cat. No. AS014, ABclonal, China; 1:5000). Target proteins were visualized via a super sensitive ECL luminescence reagent (Cat. No. AP34L025, Life-iLab, China) with a Molecular Imager ChemiDoc XRS System (Bio-Rad). A rabbit anti-FGF2 antibody (Cat. No. A0235, ABclonal, 1:100), a rabbit anti-AKT antibody (Cat. No. A17909, ABclonal, 1:1000), a rabbit anti-phospho-AKT antibody (Cat. No. AP0637, ABclonal, 1:1000), a rabbit anti-ERK1/2 antibody (Cat. No. 4695, Cell Signaling Technology, 1: 2000), a rabbit anti-phospho-ERK1/2 antibody (Cat. No. 4370, Cell Signaling Technology, 1:2000), a rabbit anti-Myc antibody (Cat. No. A1309, ABclonal, China; 1:1000), a rabbit anti-phospho-Myc-S62 antibody (Cat. No. AP0989, ABclonal, China; 1:1000), and a mouse anti-beta-Actin antibody (Cat. No. AC004, ABclonal, China; 1:2000) were used as primary antibodies. Full and uncropped western blots are presented in Supplemental File.

### ChIP

A ChIP assay kit (Cat. No. p2078, Beyotime) was applied for ChIP assay. In brief, cells were fixed using 4% PFA, and chromatin was sonicated to approximately 500–1000 bp fragments. In all, 20 μL of the sonicated chromatin was collected for input, and 180 μL of the sonicated chromatin was immunoprecipitated by a rabbit anti-ETS antibody (Cat. No. A13302, ABclonal, 1:200) or a rabbit nonimmune IgG antibody (Cat. No. AC005, ABclonal, 1:200). The protein-DNA complexes were de-bound by mixing with 5 M NaCl and incubated at 65 °C for 4 hours. After purification, DNA fragments were used for qPCR analysis. The mouse *Myc* promoter primer pair includes the following: *Myc* promoter −1378 bp region forward 5′-ACTCATGTTTCTGGTTGGT-3′, *Myc* promoter −1378 bp region reverse 5′-TTGCCTTCGTATGTGTGT-3′; *Myc* promoter -108 bp region forward 5′- GGGCGGGGAAGCGAGAG-3′, *Myc* promoter −108 bp region reverse 5′-GTCCTCGGCCGCGCAGA-3′. The mouse *Myc* exon primer pair includes the following: *Myc* exon 2 region forward 5′-GCTCTGCTCTCCATCCTA-3′, *Myc* exon 2 region reverse 5′-AGTAACTCGGTCATCATCTC-3′. Data was normalized with the nonimmune IgG.

### FACS analysis and TIME analysis

Tumor-bearing mice were sacrificed, and peripheral blood samples were collected in an anti-coagulation tube. RBCs were removed by an RBC lysis buffer (Cat. No. MA0207, Meilunbio, China). Blood cells were then washed with PBS. GFP^+^ CTCs were detected using a FACS system (FACSCanto II, BD), whereas healthy mouse blood and in vitro cultured GFP^+^ tumor cells were used as controls. Data was analyzed by Flowjo software (Version 10, BD). For humanization detection, a PE anti-mouse CD45 antibody (Cat. No. 103106, Biolegend) and a FITC anti-human CD45 antibody (Cat. No. 304006, Biolegend) were used. For TIME analysis, tumor tissue suspension was treated with an RBC lysis buffer (Cat. No. MA0207, Meilunbio, China). After washing, cell suspension was then fixed for 30 min with 4% PFA, incubated with an eFluor 780 Viability Dye (Cat. No. 65-0865-18, eBioscience) for 10 min, and incubated with various conjugated antibodies for 30 min on ice. These antibodies include: a BV510 anti-mouse CD45 antibody (Cat. No. 103138, Biolegend); a BV711 anti-mouse CD11b antibody (Cat. No. 563168, BD); an APC anti-mouse F4/80 antibody (Cat. No. 123116, Biolegend); a PE-Cy7 anti-mouse CD3 antibody (Cat. No. 25-0031-82, eBioscience); a PE anti-mouse B220 antibody (Cat. No. 12-0452-82, eBioscience); an FITC anti-CD49b antibody (Cat. No. 11-5971-82, eBioscience); an eF506 anti-human CD45 antibody (Cat. No. 69-0459-42, eBioscience); a PE-Cy7 anti-human CD14 (Cat. No. 17-4801-82, Biolegend); an APC anti-human CD56 antibody (Cat. No. 17-0567-42, eBioscience); a BV421 anti-human CD19 antibody (Cat. No. 302234, Biolegend); a BV395 anti-human CD3 antibody (Cat. No. 564001, BD). The stained cells were applied onto FACScan (BD) and analyzed by Flowjo software (Version 10, BD).

### Blood culture

Tumor-bearing mice were sacrificed, and peripheral blood samples were collected in an anti-coagulation tube. RBCs were removed by an RBC lysis buffer (Cat. No. MA0207, Meilunbio, China). Blood cells were then washed with PBS. The cell suspension was seeded onto six-well plates for 24 h, and the non-adherent cells were removed by changing the medium. Cells were cultured with 10% FBS-DMEM (Cat. No. 10099-141, Gibco; Cat. No. MA0213, Meilunbio, China) for 10 days, and stained with crystal violet (Cat. No. MA0149, Meilunbio, China) for further analysis.

### Drug treatment

A rabbit anti-mouse monoclonal VEGF-specific neutralizing antibody (BD0801) was kindly provided by Prof. Yihai Cao at the Karolinska Institute, Stockholm, Sweden [44]. The anti-VEGF neutralizing antibody at 2.5 mg/kg was intraperitoneally injected twice per week into each mouse. Lenvatinib (Cat. No. L-5400, LC Laboratories) at a dose of 30 mg/kg or Sunitinib (Cat. No. MB1229, Meilunbio, China) at a dose of 50 mg/kg was orally administrated every day for two consecutive weeks. All in vivo drug treatments starting from tumor size reached 0.5 cm^3^. For in vitro experiments, cells were starved overnight with 1% FBS-DMEM, followed by the treatment of an FGFR1 inhibitor PD166866 (Cat. No. HY-101296, MedChemExpress), an FGFR2 inhibitor alofanib (Cat. No. S8754, Selleck), an FGFR pan-inhibitor AZD4547 (Cat. No. S2801, Selleck), an ERK1/2 inhibitor SCH772984 (Cat. No. S2801S7101, Selleck), a MEK1/2 inhibitor U0126 (Cat. No. HY-12031A, MedChemExpress), an anti-VEGFR2 neutralizing antibody (Cat. No. DC101, ImClone), VEGF (Cat. No. 450-32, PEPROTECH), Sunitinib (Cat. No. MB1229, Meilunbio, China), or FGF-2 (Cat. No. 10014-HNAE, SinoBiological, China). After 48 h (or 30 min for ERK and AKTphosphorylation detections), cell lysates were collected for qPCR or western blot analysis. DMSO (Cat. No. MB2505, Meilunbio, China) or PBS was used as a control. For conditioned medium collection, NPC cells were cultured with 2% FBS-DMEM for 48 h.

### SiRNA and shRNA knockdown

*Myc* siRNA and scrambled control siRNA were purchased (GenePharma, China) and transfected into mouse ECs using liposomal transfection reagent (Cat. No. 40802ES03, YEASEN, China). The knockdown efficiency was detected by qPCR after 48 h. For shRNA experiments, sh*Scrambled* vector and sh*FGF2* vector were transfected into 5–8F cell line with GFP using a lentiviral system (GeneCopoeia Inc., USA). Briefly, 293T cells were transfected with lentiviral vectors for lentivirus production. Cells were cultured for an additional 72 h, and the conditioned media containing lentiviral particles were used for stably transfecting 5–8F NPC cell lines with polybrene (Cat. No. 40804ES76, YEASEN, China). Puromycin (Cat. No. 60210ES25, YEASEN, China) was used for selecting the successfully transfected NPC clones. The knockdown efficacy was detected by qPCR.

### Database analysis

RNA expression profiles of various cancer types TCGA data were downloaded from UCSC Xena database (https://xenabrowser.net/datapages/). Affymetrix human gene array data was downloaded from the Gene Expression Omnibus with accession numbers GSE12452 and GSE102349. R package “ggplot2” was used to perform differentially expressed genes analysis. RNA expression levels of selected genes were collected for further analysis.

### Statistical analysis

Statistical computations were performed using GraphPad Prism (GraphPad, USA). Statistical differences between two groups were determined by a two-tailed Student’s *t* test or by the Mann–Whitney test when the data distribution was not normal. *P* < 0.05 was considered statistically significant, *P* < 0.01 was very significant, and *P* < 0.001 was extremely significant. Statistical differences among multiple groups were evaluated using a one-way ANOVA test. The variance was similar between the groups that were being statistically compared. No statistical methods were used to predetermine the sample size. The data is presented as means ± standard deviation (SD).

## Supplementary information

Supplemental Figures

Supplemental Material

Reproducibility checklist

Authorship confirmation: Email collection

## Data Availability

The data that support the findings of this study are available on request from the corresponding author.
