# Supplementary material for: Lenvatinib for effectively treating antiangiogenic drug-resistant nasopharyngeal carcinoma
Source: Cell Death Dis. 2022 Aug 19;13(8):724. doi: 10.1038/s41419-022-05171-3 (PMC9391381; doi:10.1038/s41419-022-05171-3)
Supplement: Supplementary file 1 — Supplemental Figures [file 41419_2022_5171_MOESM1_ESM.pdf]

Lenvatinib for effectively treating antiangiogenic drug-resistant nasopharyngeal carcinoma

Qi Sun[1#], Yujie Wang[2#], Hong Ji[3#], Xiaoting Sun[1,4,5], Sisi Xie[1,6], Longtian Chen[6], Sen Li[1], Weifan Zeng[1], Ruibo Chen[1], Qi Tang[1], Ji Zuo[1], Likun Hou[7], Kayoko Hosaka[4] Yongtian Lu[2], Ying Liu[8*], Ying Ye[9*] and Yunlong Yang[1*]

[1]Department of Cellular and Genetic Medicine, School of Basic Medical Sciences, Fudan University, Shanghai 200032, China

[2]Department of Otolaryngology, Shenzhen Key Laboratory of Nanozymes and Translational Cancer Research, Shenzhen Institute of Translational Medicine, The First Affiliated Hospital of Shenzhen University, Shenzhen Second People's Hospital, Shenzhen, Guangdong 518035, China

[3]Department of Radiation Oncology, Jiangsu Cancer Hospital & Jiangsu Institute of Cancer Research & The Affiliated Cancer Hospital of Nanjing Medical University, Nanjing, Jiangsu, China

[4]Department of Microbiology, Tumor and Cell Biology, Karolinska Institutet, Stockholm, Sweden.

[5]Oujiang Laboratory (Zhejiang Lab for Regenerative Medicine, Vison and Brain Health), School of Pharmaceutical Science, Wenzhou Medical University, Wenzhou, P.R. China.

[6]Longyan First Hospital Affiliated to Fujian Medical University, Longyan 364000, Fujian, China

[7]Department of Pathology, Shanghai Pulmonary Hospital, Tongji University School of Medicine, Shanghai, PR China

[8]Institute of Translational Medicine, Shanghai University, 99 Shangda Road, Shanghai 200444, China

[9]Department of Oral Implantology, School and Hospital of Stomatology, Tongji University; Shanghai Engineering Research Center of Tooth Restoration and Regeneration, Shanghai, China

Keywords: lenvatinib, fibroblast growth factor-2, nasopharyngeal carcinoma, antiangiogenic drug, drug resistance

[#]These authors contributed equally.
*Corresponding authors. Galley proofs and reprint requests should be addressed to: Ying Liu, Ph.D., Institute of Translational Medicine, Shanghai University, 99 Shangda Road, Shanghai 200444, China. Email: liuchanger1984@163.com; Ying Ye, D.M.D, Ph.D., Department of Oral Implantology, School and Hospital of Stomatology, Tongji University; Shanghai Engineering Research Center of Tooth Restoration and Regeneration, Shanghai, China. Email: ying.ye@tongji.edu.cn; Yunlong Yang, Ph.D., Department of Cellular and Genetic Medicine, School of Basic Medical Sciences,

Fudan University, Shanghai 200032, P.R. China. Tel: (+86)-21-5423 7311, E-mail: yunlongyang@fudan.edu.cn

Running title: Lenvatinib overcomes NPC antiangiogenic drug resistance

Supplemental Figure Legends

Fig.S1

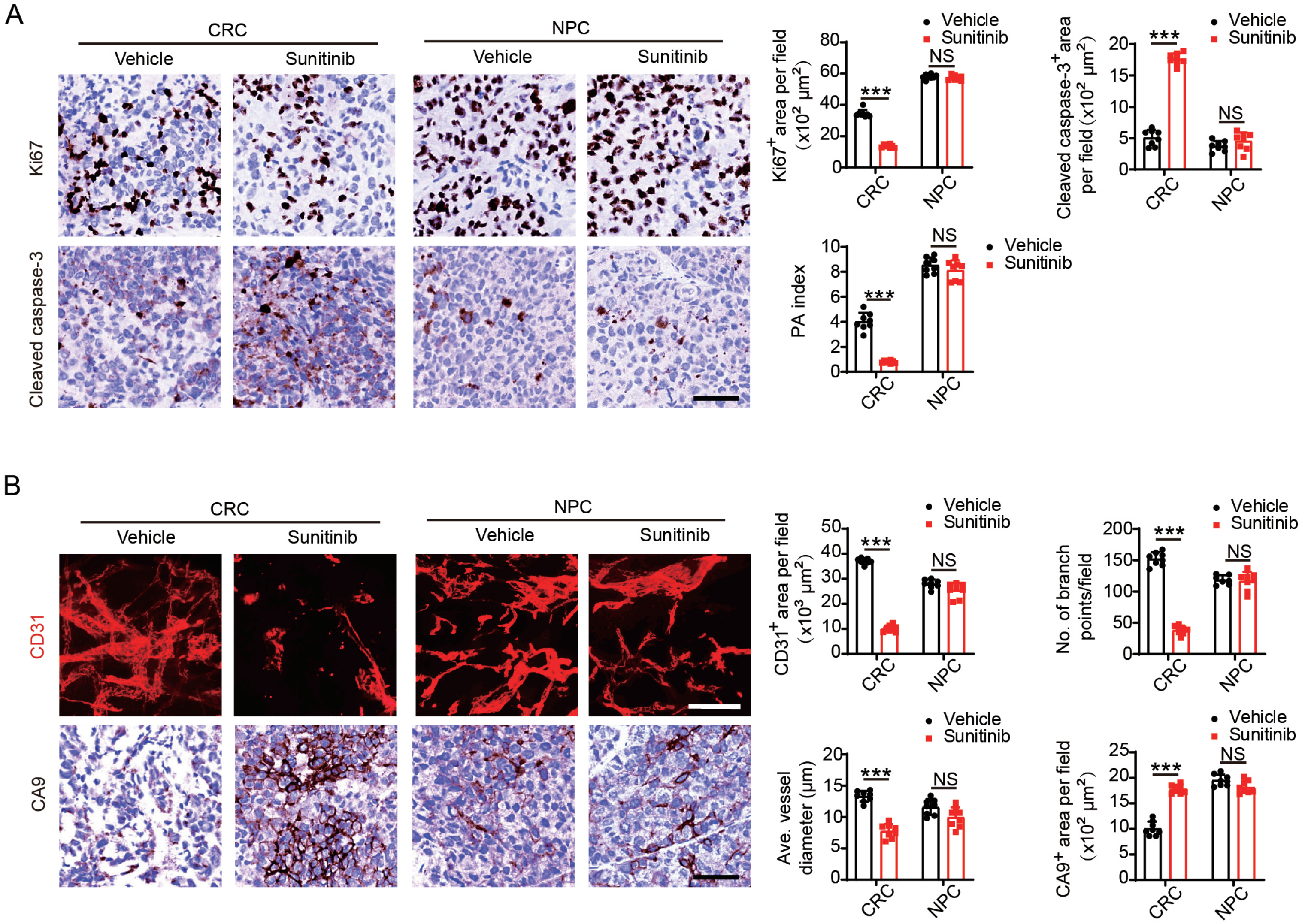
A
CRC
NPC
Vehicle
Sunitinib
Ki67
Cleaved caspase-3
Ki67+ area per field (x10² μm²)
Cleaved caspase-3+ area per field (x10² μm²)
PA index
***
NS
CRC
NPC
B
CD31
CA9
CD31+ area per field (x10³ μm²)
No. of branch points/field
Ave. vessel diameter (μm)
CA9+ area per field (x10² μm²)

Figure S1. Tumor and vasculature responses to sunitinib in CRC and NPC.

(A) Representative micrographs of $Ki67^+$ proliferative cells and cleaved caspase-3^+ apoptotic cells in vehicle- or sunitinib-treated CRC and NPC tumors. Scale bar=50 μm. Quantification of $Ki67^+$, cleaved caspase-3^+ signals, and PA index in vehicle- or sunitinib-treated CRC and NPC tumors (n=8 random fields per group). (B) Representative micrographs of $CD31^+$ microvessels and $CA9^+$ hypoxic areas in vehicle- or sunitinib-treated CRC and NPC tumors. Scale bar in upper panel=100 μm, scale bar in lower panel=50 μm. Quantification of $CD31^+$ tumor vessel parameters and $CA9^+$ signals in vehicle- or sunitinib-treated CRC and NPC tumors (n=8 random fields per group). ***p<0.001. NS=not significant. Data presented as mean±SD.

Fig. S2

A

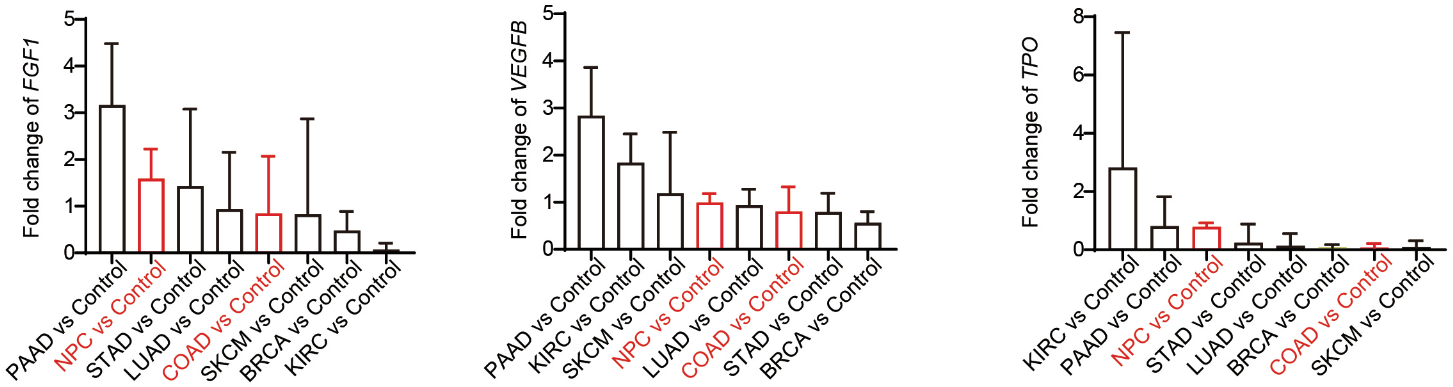

Fold change of FGF1
5
4
3
2
1
0
PAAD vs Control
NPC vs Control
STAD vs Control
LUAD vs Control
COAD vs Control
SKCM vs Control
BRCA vs Control
KIRC vs Control
Fold change of VEGFB
5
4
3
2
1
0
PAAD vs Control
KIRC vs Control
SKCM vs Control
NPC vs Control
LUAD vs Control
COAD vs Control
STAD vs Control
BRCA vs Control
Fold change of TPO
8
6
4
2
0
KIRC vs Control
PAAD vs Control
NPC vs Control
STAD vs Control
LUAD vs Control
BRCA vs Control
COAD vs Control
SKCM vs Control

Figure S2. Expression levels of angiogenic factors in various types of cancer.

(A) Transcriptomic expression levels of angiogenic factors, including *FGF1*, *VEGFB*, and *TPO* in human KIRC tissues, COAD tissues, NPC tissues, STAD tissues, BRCA tissues, SKCM tissues, and their adjacent healthy tissues. KIRC, kidney renal clear cell carcinoma; COAD, colon adenocarcinoma; STAD, stomach adenocarcinoma; PAAD, pancreatic adenocarcinoma; LUAD, lung adenocarcinoma; BRCA, breast invasive carcinoma; SKCM, skin cutaneous melanoma.

Fig.S3

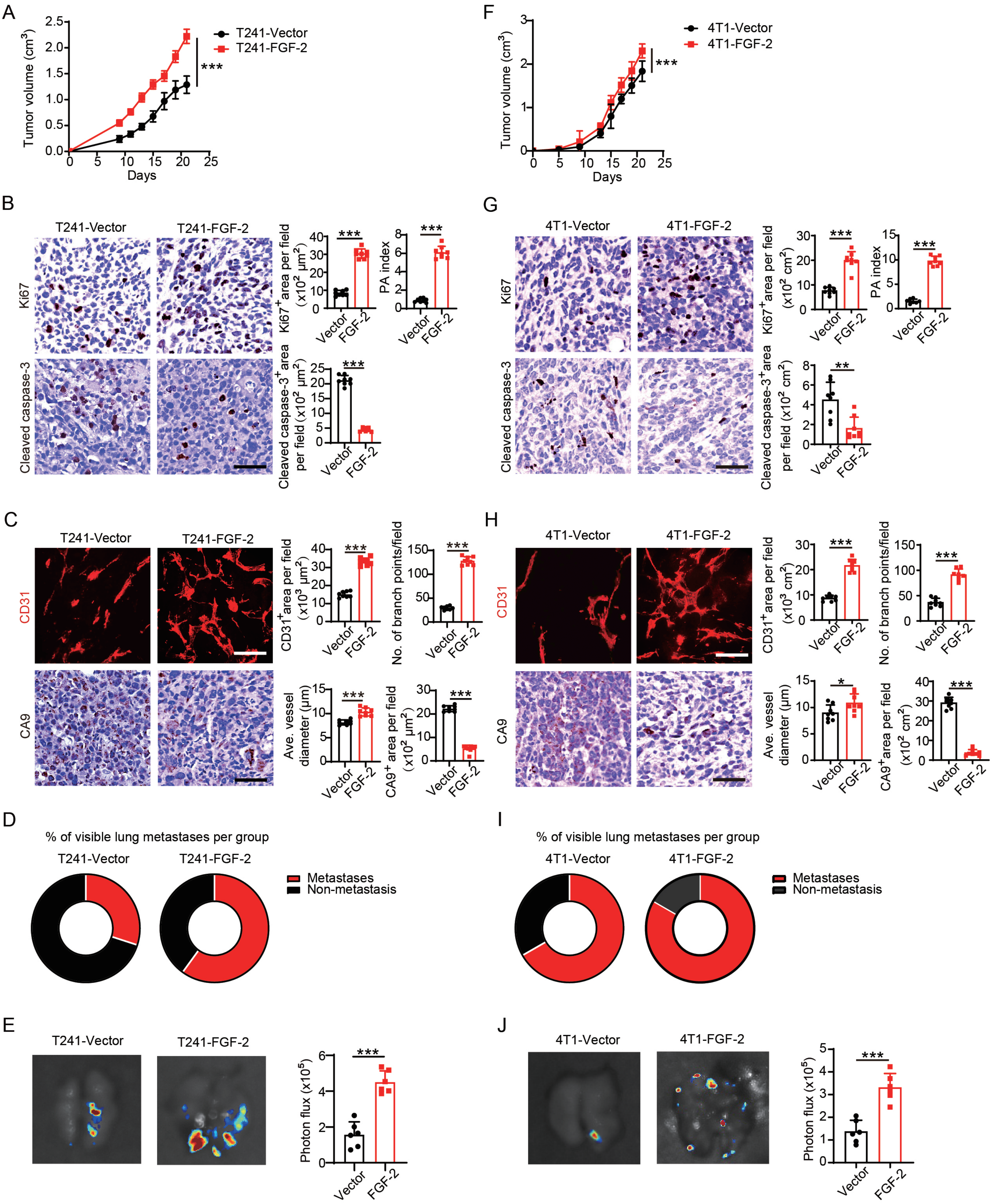

A
Tumor volume (cm3)
Days
T241-Vector
T241-FGF-2
***
F
4T1-Vector
4T1-FGF-2
B
T241-Vector
T241-FGF-2
Ki67
Cleaved caspase-3
Ki67+area per field (x10² μm²)
PA index
Cleaved caspase-3+area per field (x10² μm²)
Vector
FGF-2
G
4T1-Vector
4T1-FGF-2
**
C
CD31
CA9
CD31+area per field (x10³ μm²)
No. of branch points/field
Ave. vessel diameter (μm)
CA9+ area per field (x10² μm²)
H
*
D
% of visible lung metastases per group
Metastases
Non-metastasis
I
E
Photon flux (x10⁵)
J

Figure S3. Tumor cell-derived FGF-2 promotes angiogenesis and tumor metastasis in mouse models.

(A and F) Tumor growth of vector- or FGF-2-transfected T241 or 4T1 tumors. (B and G) Representative micrographs of Ki67^{+} proliferative cells and cleaved caspase-3^{+} apoptotic cells in T241 or 4T1 tumors. Scale bar=50 μm. Quantification of Ki67^{+}, cleaved caspase-3^{+} signals, and PA index in NPC (n=8 random fields per group). (C and H) Representative micrographs of CD31^{+} microvessels and CA9^{+} hypoxic areas in T241 or 4T1 tumors. Scale bar in upper panel=100 μm, scale bar in lower panel=50 μm. Quantification of CD31^{+} tumor vessel parameters and CA9^{+} signals in T241 or 4T1 tumors. (n=8 random fields per group). (D and I) Quantification of pulmonary metastasis proportion in T241- or 4T1-bearing mice (n=6 mice per group). (E and J) Representative graphs of EGFP^{+} metastatic signals in the lung. Quantification of photon flux (n=6 lungs per group). *$p<0.05$; **$p<0.01$; ***$p<0.001$. NS=not significant. Data presented as mean±SD.

Fig. S4

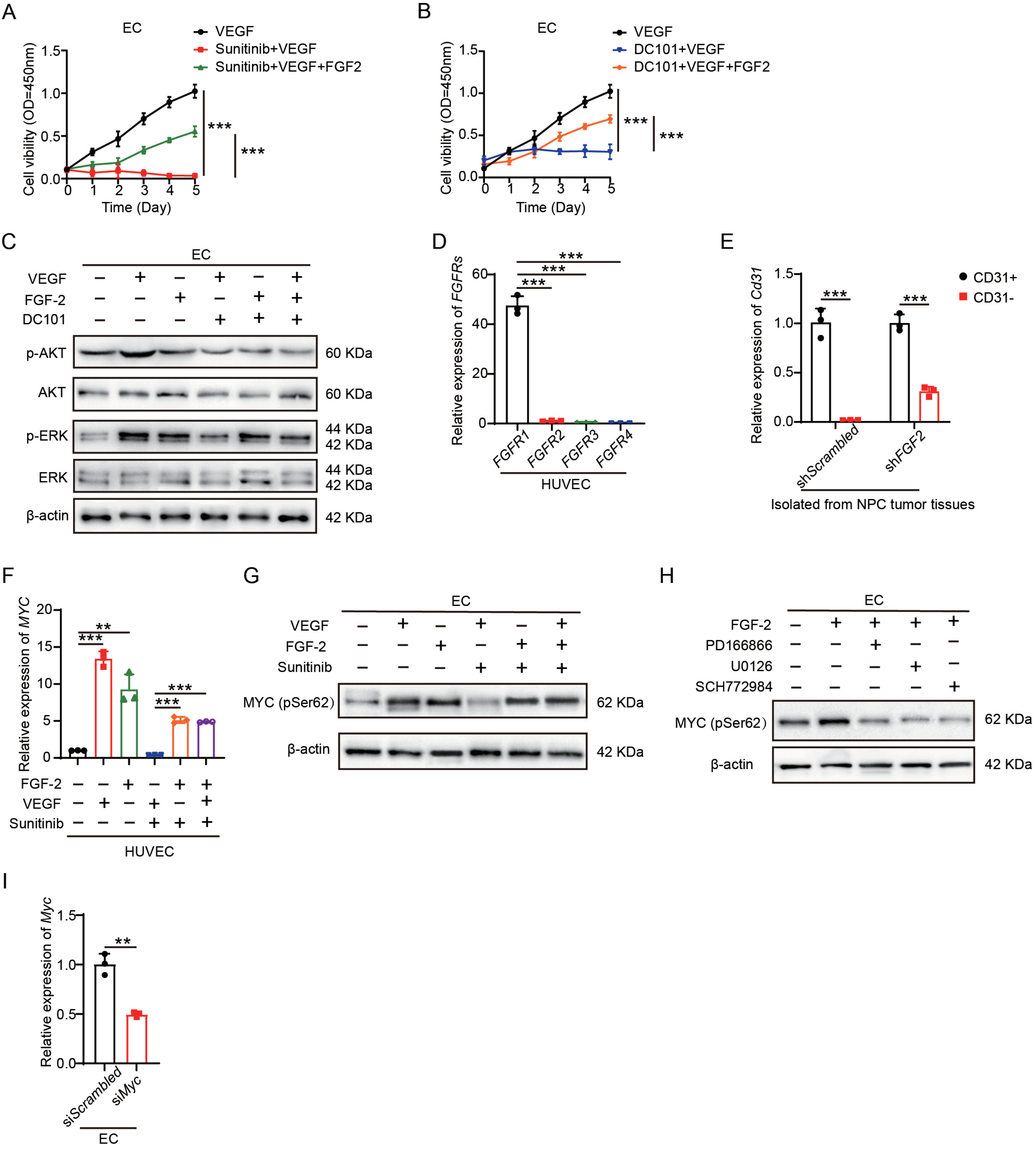

A
EC
VEGF
Sunitinib+VEGF
Sunitinib+VEGF+FGF2
Cell vibility (OD=450nm)
Time (Day)
***
***
B
EC
VEGF
DC101+VEGF
DC101+VEGF+FGF2
Cell vibility (OD=450nm)
Time (Day)
***
***
C
EC
VEGF
FGF-2
DC101
p-AKT
AKT
p-ERK
ERK
β-actin
60 KDa
60 KDa
44 KDa
42 KDa
44 KDa
42 KDa
42 KDa
D
Relative expression of FGFRs
FGFR1
FGFR2
FGFR3
FGFR4
HUVEC
E
Relative expression of Cd31
CD31+
CD31-
shScrambled
shFGF2
Isolated from NPC tumor tissues
F
Relative expression of MYC
FGF-2
VEGF
Sunitinib
HUVEC
G
EC
VEGF
FGF-2
Sunitinib
MYC (pSer62)
β-actin
62 KDa
42 KDa
H
EC
FGF-2
PD166866
U0126
SCH772984
MYC (pSer62)
β-actin
62 KDa
42 KDa
I
Relative expression of Myc
siScrambled
siMyc
EC

Figure S4. FGF-2 elevates phosphorylation of ERK and MYC in ECs.

(A and B) Cell growth of VEGF-treated ECs receiving with or without AAD or FGF-2 (n=5 samples per group). (C) Vehicle- or VEGF-treated ECs were challenged with or without VEGFR2 neutralizing antibody or FGF-2. Phosphorylation of AKT and ERK in ECs was detected. β-actin marks the loading level in each lane (n=3 samples per group). (D) QPCR quantification of *FGFR1*, *FGFR2*, *FGFR3*, and *FGFR4* mRNA levels in human ECs (n=3 samples per group). (E) QPCR quantification of *CD31* mRNA levels in isolated $CD31^+$ ECs from scramble- or *FGF2* shRNA-transfected NPC tumor tissues (n=3 samples per group). (F) QPCR quantification of *MYC* mRNA levels in various groups of human ECs (n=3 samples per group). (G) Vehicle- or VEGF-treated ECs were treated with or without AAD or FGF-2. MYC phosphorylation in ECs was detected. β-actin marks the loading level in each lane (n=3 samples per group). (H) Vehicle- or FGF-2-treated ECs were challenged with or without various inhibitors. Phosphorylation of MYC in ECs was detected. β-actin marks the loading level in each lane (n=3 samples per group). (I) QPCR quantification of *Myc* mRNA levels in scramble- or *Myc* siRNA-transfected ECs (n=3 samples per group). **$p<0.01$; ***$p<0.001$. NS=not significant. Data presented as mean±SD.

Fig. S5

A

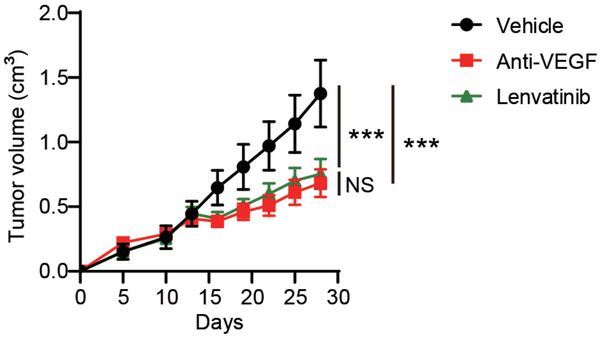

Tumor volume (cm3)
2.0
1.5
1.0
0.5
0.0
0
5
10
15
20
25
30
Days
***
***
NS
Vehicle
Anti-VEGF
Lenvatinib

Figure S5. Similar efficacy of anti-VEGF and lenvatinib in CRC xenografts.

(A) Tumor growth was measured in vehicle-, anti-VEGF-, and lenvatinib-treated CRC tumors (n=6 samples per group). ***$p<0.001$. NS=not significant. Data presented as mean±SD.

Fig.S6

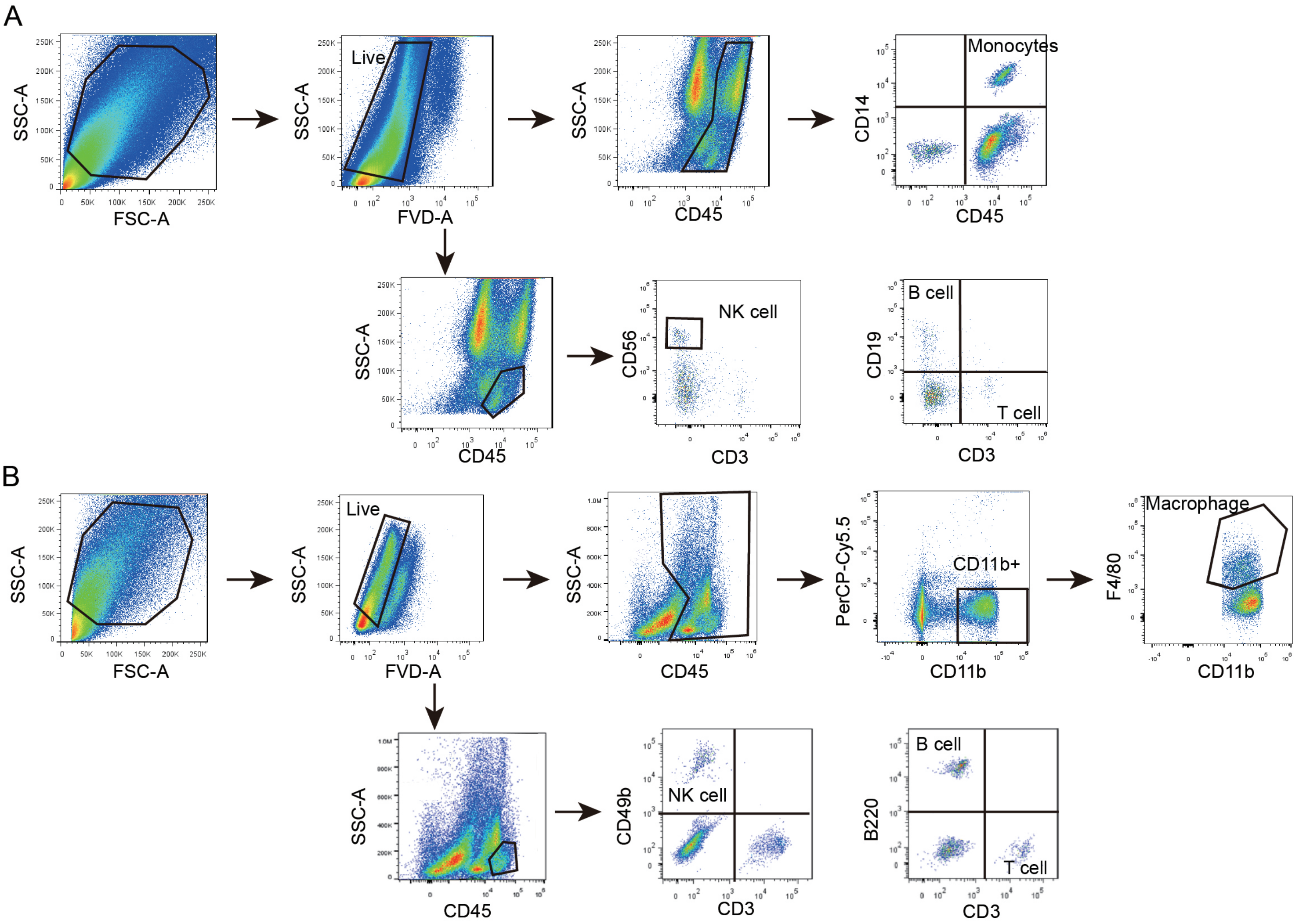

A
SSC-A
FSC-A
Live
FVD-A
CD45
Monocytes
CD14
NK cell
CD56
CD3
B cell
CD19
T cell
B
PerCP-Cy5.5
CD11b+
CD11b
Macrophage
F4/80
CD49b
B220

Figure S6. Gating strategy for FACS analysis of immune landscape in NPC in humanized NSG mice.

(A) Representative FACS profiles showing gating strategy of $hCD45^+$ $hCD14^+$ population, $hCD45^+$ $hCD19^+$ population, $hCD45^+$ $hCD3^+$ population, and $hCD45^+$ $hCD56^+$ population in the NPC TME. (B) Representative FACS profiles showing gating strategy of $mCD45^+$ $mCD11b^+$ $mF4/80^+$ population, $mCD45^+$ $mB220^+$ population, $mCD45^+$ $mCD3^+$ population, and $mCD45^+$ $mCD49b^+$ population.
