# Supplementary figures and images for: Lenvatinib for effectively treating antiangiogenic drug-resistant nasopharyngeal carcinoma
Source: Cell Death Dis. 2022 Aug 19;13(8):724. doi: 10.1038/s41419-022-05171-3 (PMC9391381; doi:10.1038/s41419-022-05171-3)

Fig.4C

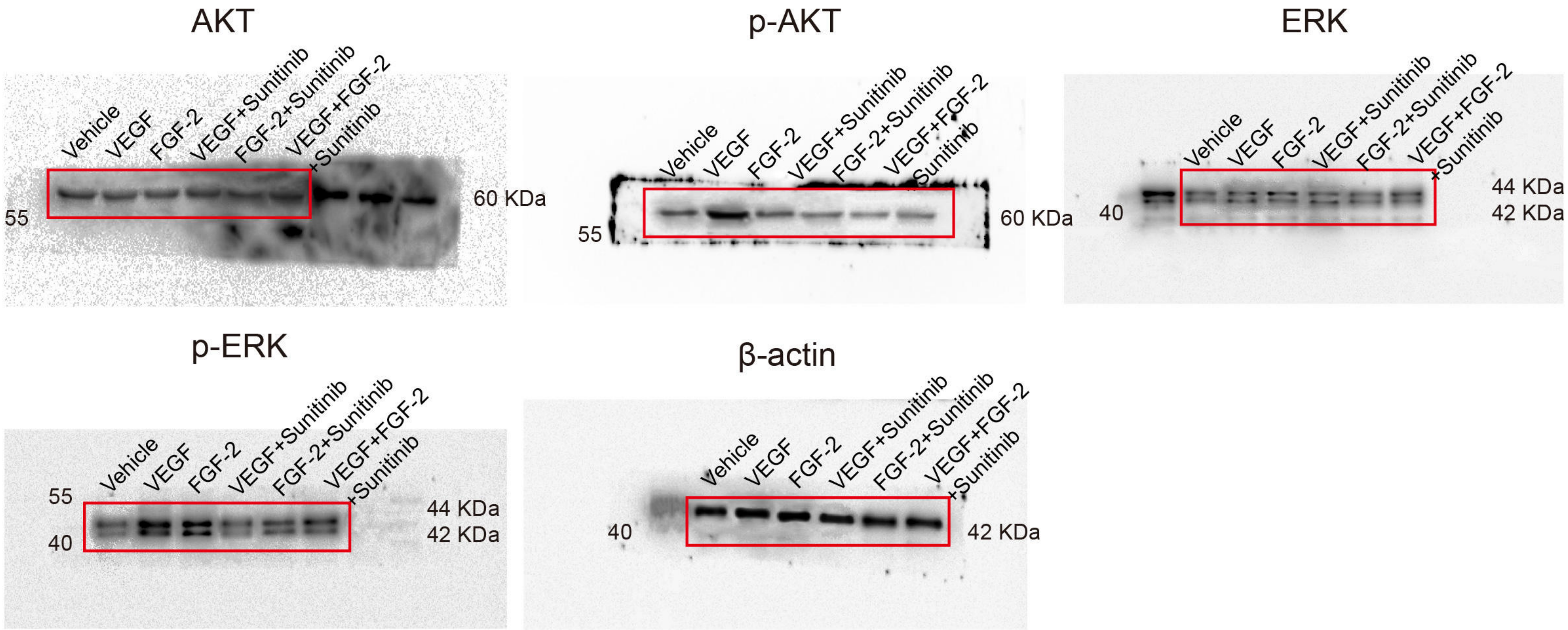

Fig.4E

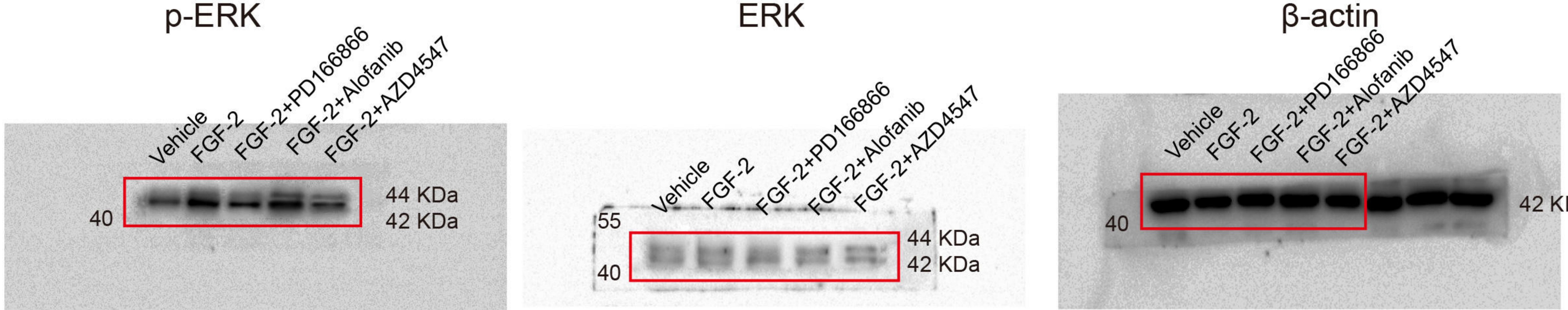

Fig.4J

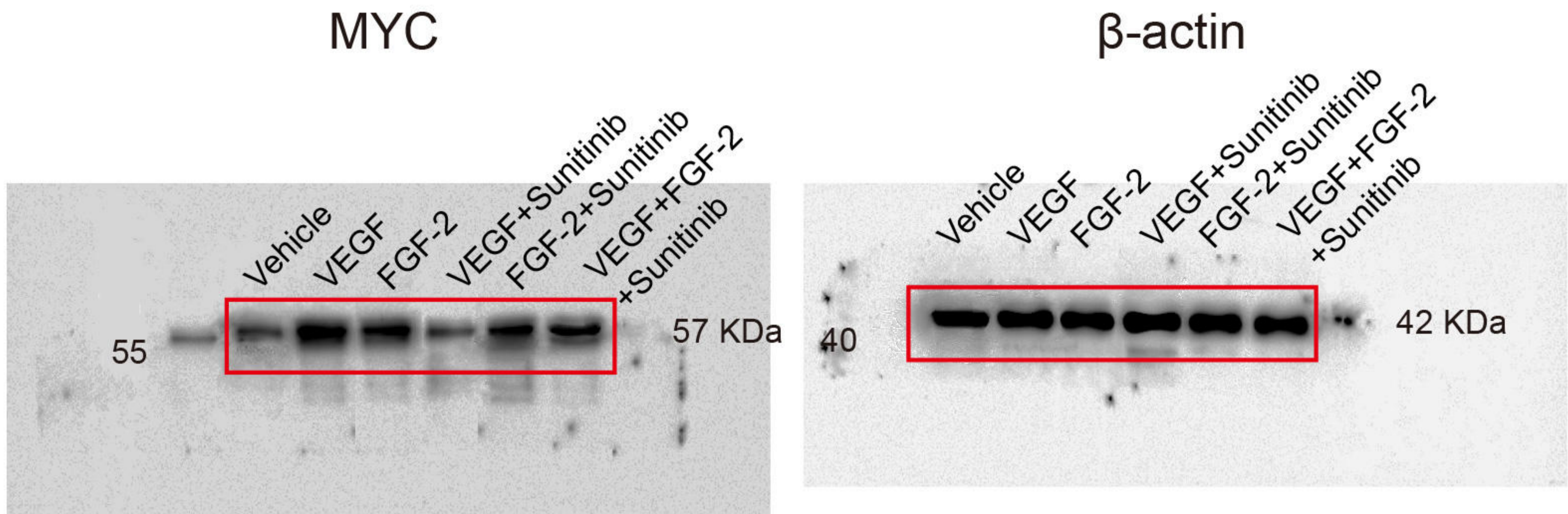

Fig.6I

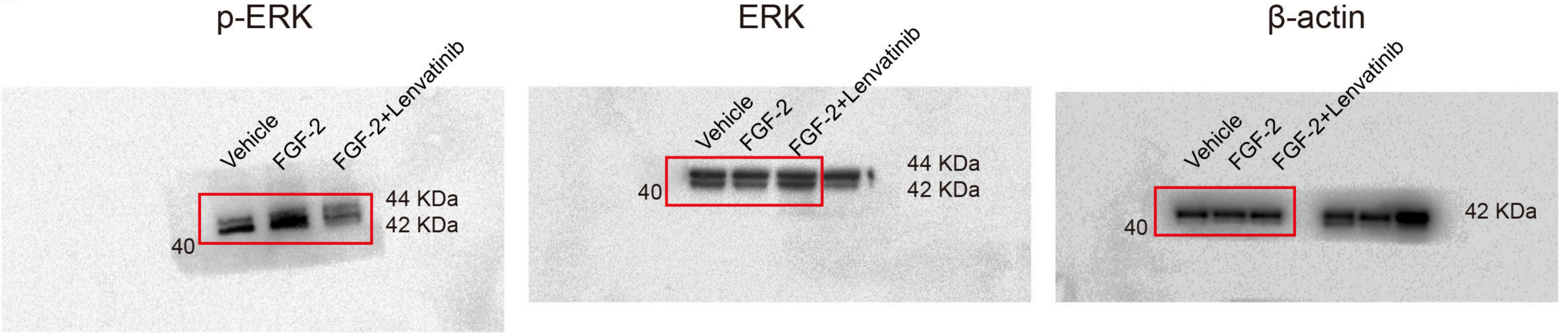

Fig.S4C

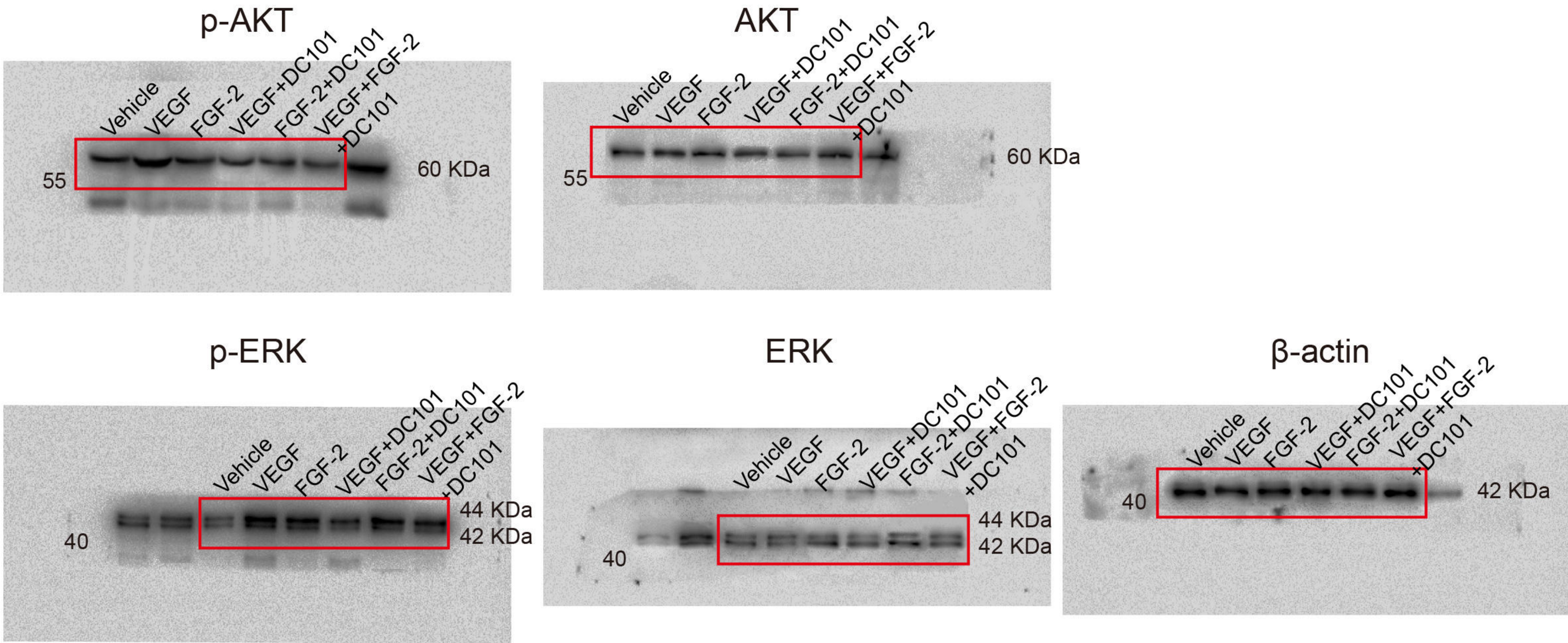

Fig.S4G

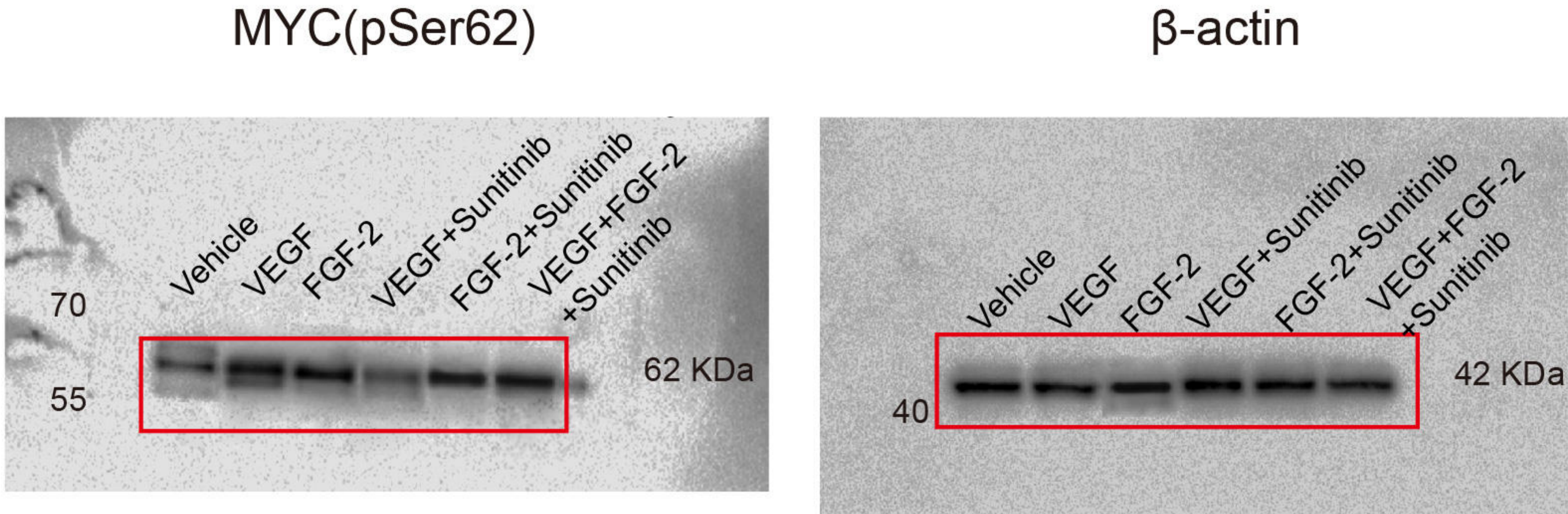

Fig.S4H

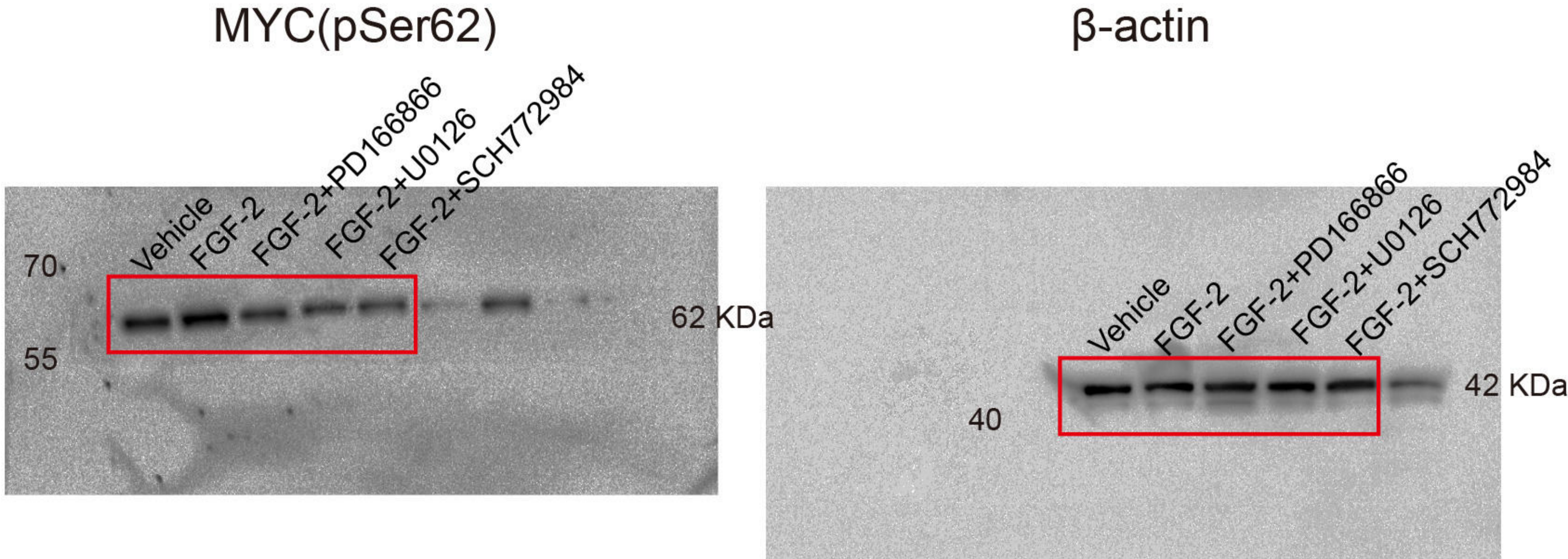

Supplement: Supplementary file 2 — Supplemental Material [file 41419_2022_5171_MOESM2_ESM.pdf]
