# Supplementary material for: Lenvatinib for effectively treating antiangiogenic drug-resistant nasopharyngeal carcinoma
Source: Cell Death Dis. 2022 Aug 19;13(8):724. doi: 10.1038/s41419-022-05171-3 (PMC9391381; doi:10.1038/s41419-022-05171-3)
Supplement: Supplementary file 4 — Authorship confirmation: Email collection [file 41419_2022_5171_MOESM4_ESM.pdf]

Subject:	CDDIS-22-0531RR authorship confirmation
From:	"Yunlong Yang" <yunlongyang@fudan.edu.cn> Aug 4, 2022 10:18:49 PM
To:	"孙琪" <19111010006@fudan.edu.cn>, "王雨洁" <wang_maggie_223@163.com>, dr_jihong@163.com, xiaoting <xiaoting9203@126.com>, "谢思思" <18111010026@fudan.edu.cn>, ltchen@163.com, "李森" <20211230014@fudan.edu.cn>, "曾苇凡" <17301010015@fudan.edu.cn>, "陈瑞波" <17301010009@fudan.edu.cn>, 15301050249@fudan.edu.cn, "左伋" <jzuo@shmu.edu.cn>, hlk9575@163.com, "Kayoko.Hosaka" <Kayoko.Hosaka@ki.se>, "卢永田" <luyongtian@263.net>, liuchanger1984@163.com, "叶颖" <ying.ye@tongji.edu.cn>

Dear co-authors,

Before our CDDIS paper is officially accepted, I need you to send an official confirmation of the current authorship.

Please reply to this email indicating you agree with the current authorship.

e.g. "I agree with the above mentioned authorship.

Your name in English"

Current authorship:

Qi Sun1#, Yujie Wang2#, Hong Ji3#, Xiaoting Sun1,4,5, Sisi Xie1,6, Longtian Chen6, Sen Li1, Weifan Zeng1, Ruibo Chen1, Qi Tang1, Ji Zuo1, Likun Hou7, Kayoko Hosaka4 Yongtian Lu2, Ying Liu8*, Ying Ye9* and Yunlong Yang1*

1Department of Cellular and Genetic Medicine, School of Basic Medical Sciences, Fudan University, Shanghai 200032, China

2Department of Otolaryngology, Shenzhen Key Laboratory of Nanozymes and Translational Cancer Research, Shenzhen Institute of Translational Medicine, The First Affiliated Hospital of Shenzhen University, Shenzhen Second People’s Hospital, Shenzhen, Guangdong 518035, China

3Department of Radiation Oncology, Jiangsu Cancer Hospital & Jiangsu Institute of Cancer Research & The Affiliated Cancer Hospital of Nanjing Medical University, Nanjing, Jiangsu, China

4Department of Microbiology, Tumor and Cell Biology, Karolinska Institutet, Stockholm, Sweden.

5Oujiang Laboratory (Zhejiang Lab for Regenerative Medicine, Vison and Brain Health), School of Pharmaceutical Science, Wenzhou Medical University, Wenzhou, P.R. China.

6Longyan First Hospital Affiliated to Fujian Medical University, Longyan 364000, Fujian, China

7Department of Pathology, Shanghai Pulmonary Hospital, Tongji University School of Medicine, Shanghai, PR China

8Institute of Translational Medicine, Shanghai University, 99 Shangda Road, Shanghai 200444, China

9Department of Oral Implantology, School and Hospital of Stomatology, Tongji University; Shanghai Engineering Research Center of Tooth Restoration and Regeneration, Shanghai, China

Thank you so much for your help.

Best regards.

Yunlong Yang

Fudan University

Subject:	Re: CDDIS-22-0531RR authorship confirmation
From:	"孙琪" <19111010006@fudan.edu.cn> Aug 5, 2022 8:05:00 PM
To:	"Yunlong Yang" <yunlongyang@fudan.edu.cn>

I agree with the above mentioned authorship.
Qi Sun

-----原始邮件-----
发件人:"Yunlong Yang" <yunlongyang@fudan.edu.cn>
发送时间:2022-08-04 22:18:49 (星期四)
收件人: "孙琪" <19111010006@fudan.edu.cn>, "王雨洁" <wang_maggie_223@163.com>, dr_jihong@163.com, xiaoting <xiaoting9203@126.com>, "谢思思" <18111010026@fudan.edu.cn>, ltchen@163.com, "李森" <20211230014@fudan.edu.cn>, "曾苇凡" <17301010015@fudan.edu.cn>, "陈瑞波" <17301010009@fudan.edu.cn>, 15301050249@fudan.edu.cn, "左伋" <jzuo@shmu.edu.cn>, hlk9575@163.com, "Kayoko.Hosaka" <Kayoko.Hosaka@ki.se>, "卢永田" <luyongtian@263.net>, liuchanger1984@163.com, "叶颖" <ying.ye@tongji.edu.cn>
抄送:
主题: CDDIS-22-0531RR authorship confirmation

Dear co-authors,

Before our CDDIS paper is officially accepted, I need you to send an official confirmation of the current authorship.

Please reply to this email indicating you agree with the current authorship.

e.g. "I agree with the above mentioned authorship.

Your name in English"

Current authorship:

Qi Sun1#, Yujie Wang2#, Hong Ji3#, Xiaoting Sun1,4,5, Sisi Xie1,6, Longtian Chen6, Sen Li1, Weifan Zeng1, Ruibo Chen1, Qi Tang1, Ji Zuo1, Likun Hou7, Kayoko Hosaka4 Yongtian Lu2, Ying Liu8*, Ying Ye9* and Yunlong Yang1*

1Department of Cellular and Genetic Medicine, School of Basic Medical Sciences, Fudan University, Shanghai 200032, China

2Department of Otolaryngology, Shenzhen Key Laboratory of Nanozymes and Translational Cancer Research, Shenzhen Institute of Translational Medicine, The First Affiliated Hospital of Shenzhen University, Shenzhen Second People's Hospital, Shenzhen, Guangdong 518035, China

3Department of Radiation Oncology, Jiangsu Cancer Hospital & Jiangsu Institute of Cancer Research & The Affiliated Cancer Hospital of Nanjing Medical University, Nanjing, Jiangsu, China

4Department of Microbiology, Tumor and Cell Biology, Karolinska Institutet, Stockholm, Sweden.

5Oujiang Laboratory (Zhejiang Lab for Regenerative Medicine, Vison and Brain Health), School of Pharmaceutical Science, Wenzhou Medical University, Wenzhou, P.R. China.

6Longyan First Hospital Affiliated to Fujian Medical University, Longyan 364000, Fujian, China

7Department of Pathology, Shanghai Pulmonary Hospital, Tongji University School of Medicine, Shanghai, PR China

8Institute of Translational Medicine, Shanghai University, 99 Shangda Road, Shanghai 200444, China

9Department of Oral Implantology, School and Hospital of Stomatology, Tongji University; Shanghai Engineering Research Center of Tooth Restoration and Regeneration, Shanghai, China

Thank you so much for your help.

Best regards.

Yunlong Yang

Fudan University

Subject:	Re: CDDIS-22-0531RR authorship confirmation	
From:	"王雨洁" <wang_maggie_223@163.com>	Aug 4, 2022 10:23:56 PM
To:	"Yunlong Yang" <yunlongyang@fudan.edu.cn>	

I agree with the above mentioned authorship.

Yujie Wang

---- Replied Message ----

From	Yunlong Yang<yunlongyang@fudan.edu.cn>
Date	08/04/2022 22:18
To	孙琪<19111010006@fudan.edu.cn>,<br>王雨洁<wang_maggie_223@163.com>,<br>dr_jihong<dr_jihong@163.com>,<br>xiaoting <xiaoting9203@126.com>,<br>谢思思<18111010026@fudan.edu.cn>,<br>ltchen<ltchen@163.com>,<br>李森<20211230014@fudan.edu.cn>,<br>曾苇凡<17301010015@fudan.edu.cn>,<br>陈瑞波<17301010009@fudan.edu.cn>,<br>15301050249<15301050249@fudan.edu.cn>,<br>左伋<jzuo@shmu.edu.cn>,<br>hlk9575<hlk9575@163.com>,<br>Kayoko.Hosaka<kayoko.hosaka@ki.se>,<br>卢永田<luyongtian@263.net>,<br>liuchanger1984<liuchanger1984@163.com>,<br>叶颖<ying.ye@tongji.edu.cn>
Subject	CDDIS-22-0531RR authorship confirmation

Dear co-authors,

Before our CDDIS paper is officially accepted, I need you to send an official confirmation of the current authorship.

Please reply to this email indicating you agree with the current authorship.

e.g. "I agree with the above mentioned authorship.

Your name in English"

Current authorship:

Qi Sun1#, Yujie Wang2#, Hong Ji3#, Xiaoting Sun1,4,5, Sisi Xie1,6, Longtian Chen6, Sen Li1, Weifan Zeng1, Ruibo Chen1, Qi Tang1, Ji Zuo1, Likun Hou7, Kayoko Hosaka4 Yongtian Lu2, Ying Liu8*, Ying Ye9* and Yunlong Yang1*

1Department of Cellular and Genetic Medicine, School of Basic Medical Sciences, Fudan University, Shanghai 200032, China

2Department of Otolaryngology, Shenzhen Key Laboratory of Nanozymes and Translational Cancer Research, Shenzhen Institute of Translational Medicine, The First Affiliated Hospital of Shenzhen University, Shenzhen Second People's Hospital, Shenzhen, Guangdong 518035, China

3Department of Radiation Oncology, Jiangsu Cancer Hospital & Jiangsu Institute of Cancer Research & The Affiliated Cancer Hospital of Nanjing Medical University, Nanjing, Jiangsu, China

4Department of Microbiology, Tumor and Cell Biology, Karolinska Institutet, Stockholm, Sweden.

5Oujiang Laboratory (Zhejiang Lab for Regenerative Medicine, Vison and Brain Health), School of Pharmaceutical Science, Wenzhou Medical University, Wenzhou, P.R. China.

6Longyan First Hospital Affiliated to Fujian Medical University, Longyan 364000, Fujian, China

7Department of Pathology, Shanghai Pulmonary Hospital, Tongji University School of Medicine, Shanghai, PR China

8Institute of Translational Medicine, Shanghai University, 99 Shangda Road, Shanghai 200444, China

9Department of Oral Implantology, School and Hospital of Stomatology, Tongji University; Shanghai Engineering Research Center of Tooth Restoration and Regeneration, Shanghai, China

Thank you so much for your help.

Best regards.

Yunlong Yang

Fudan University

Subject:	Re: CDDIS-22-0531RR authorship confirmation	
From:	I <dr_jihong@163.com>	Aug 4, 2022 10:26:14 PM
To:	"杨云龙" <yunlongyang@fudan.edu.cn>	

Dear Yunlong,

I agree with the above mentioned authorship. Thank you.

Hong Ji

I
邮箱：dr_jihong@163.com

---- Replied Message ----

From	Yunlong Yang<yunlongyang@fudan.edu.cn>
Date	08/04/2022 22:18
To	孙琪<19111010006@fudan.edu.cn>,<br>王雨洁<wang_maggie_223@163.com>,<br>dr_jihong<dr_jihong@163.com>,<br>xiaoting <xiaoting9203@126.com>,<br>谢思思<18111010026@fudan.edu.cn>,<br>ltchen<ltchen@163.com>,<br>李森<20211230014@fudan.edu.cn>,<br>曾苇凡<17301010015@fudan.edu.cn>,<br>陈瑞波<17301010009@fudan.edu.cn>,<br>15301050249<15301050249@fudan.edu.cn>,<br>左伋<jzuo@shmu.edu.cn>,<br>hlk9575<hlk9575@163.com>,<br>Kayoko.Hosaka<kayoko.hosaka@ki.se>,<br>卢永田<luyongtian@263.net>,<br>liuchanger1984<liuchanger1984@163.com>,<br>叶颖<ying.ye@tongji.edu.cn>
Subject	CDDIS-22-0531RR authorship confirmation

Dear co-authors,

Before our CDDIS paper is officially accepted, I need you to send an official confirmation of the current authorship.

Please reply to this email indicating you agree with the current authorship.

e.g. "I agree with the above mentioned authorship.

Your name in English"

Current authorship:

Qi Sun1#, Yujie Wang2#, Hong Ji3#, Xiaoting Sun1,4,5, Sisi Xie1,6, Longtian Chen6, Sen Li1, Weifan Zeng1, Ruibo Chen1, Qi Tang1, Ji Zuo1, Likun Hou7, Kayoko Hosaka4 Yongtian Lu2, Ying Liu8*, Ying Ye9* and Yunlong Yang1*

1Department of Cellular and Genetic Medicine, School of Basic Medical Sciences, Fudan University, Shanghai 200032, China

2Department of Otolaryngology, Shenzhen Key Laboratory of Nanozymes and Translational Cancer Research, Shenzhen Institute of Translational Medicine, The First Affiliated Hospital of Shenzhen University, Shenzhen Second People's Hospital, Shenzhen, Guangdong 518035, China

3Department of Radiation Oncology, Jiangsu Cancer Hospital & Jiangsu Institute of Cancer Research & The Affiliated Cancer Hospital of Nanjing Medical University, Nanjing, Jiangsu, China

4Department of Microbiology, Tumor and Cell Biology, Karolinska Institutet, Stockholm, Sweden.

5Oujiang Laboratory (Zhejiang Lab for Regenerative Medicine, Vison and Brain Health), School of Pharmaceutical Science, Wenzhou Medical University, Wenzhou, P.R. China.

6Longyan First Hospital Affiliated to Fujian Medical University, Longyan 364000, Fujian, China

7Department of Pathology, Shanghai Pulmonary Hospital, Tongji University School of Medicine, Shanghai, PR China

8Institute of Translational Medicine, Shanghai University, 99 Shangda Road, Shanghai 200444, China

9Department of Oral Implantology, School and Hospital of Stomatology, Tongji University; Shanghai Engineering Research Center of Tooth Restoration and Regeneration, Shanghai, China

Thank you so much for your help.

Best regards.

Yunlong Yang

Fudan University

Subject:	回复：CDDIS-22-0531RR authorship confirmation
From:	"孙筱婷" <xiaoting9203@126.com> Aug 4, 2022 10:21:49 PM
To:	yunlongyang <yunlongyang@fudan.edu.cn>

I agree with the above mentioned authorship.

Xiaoting Sun

---- 回复的原邮件 ----

发件人	Yunlong Yang<yunlongyang@fudan.edu.cn>
日期	2022年08月04日 22:18
收件人	孙琪<19111010006@fudan.edu.cn>、王雨洁<wang_maggie_223@163.com>、dr_jihong@163.com<dr_jihong@163.com>、xiaoting<xiaoting9203@126.com>、谢思思<18111010026@fudan.edu.cn>、ltchen@163.com<ltchen@163.com>、李森<20211230014@fudan.edu.cn>、曾苇凡<17301010015@fudan.edu.cn>、陈瑞波<17301010009@fudan.edu.cn>、15301050249@fudan.edu.cn<15301050249@fudan.edu.cn>、左伋<jzuo@shmu.edu.cn>、hlk9575@163.com<hlk9575@163.com>、Kayoko.Hosaka<Kayoko.Hosaka@ki.se>、卢永田<luyongtian@263.net>、liuchanger1984@163.com<liuchanger1984@163.com>、叶颖<ying.ye@tongji.edu.cn>
主题	CDDIS-22-0531RR authorship confirmation

Dear co-authors,

Before our CDDIS paper is officially accepted, I need you to send an official confirmation of the current authorship.

Please reply to this email indicating you agree with the current authorship.

e.g. "I agree with the above mentioned authorship.

Your name in English"

Current authorship:

Qi Sun1#, Yujie Wang2#, Hong Ji3#, Xiaoting Sun1,4,5, Sisi Xie1,6, Longtian Chen6, Sen Li1, Weifan Zeng1, Ruibo Chen1, Qi Tang1, Ji Zuo1, Likun Hou7, Kayoko Hosaka4 Yongtian Lu2, Ying Liu8*, Ying Ye9* and Yunlong Yang1*

1Department of Cellular and Genetic Medicine, School of Basic Medical Sciences, Fudan University, Shanghai 200032, China

2Department of Otolaryngology, Shenzhen Key Laboratory of Nanozymes and Translational Cancer Research, Shenzhen Institute of Translational Medicine, The First Affiliated Hospital of Shenzhen University, Shenzhen Second People's Hospital, Shenzhen, Guangdong 518035, China

3Department of Radiation Oncology, Jiangsu Cancer Hospital & Jiangsu Institute of Cancer Research & The Affiliated Cancer Hospital of Nanjing Medical University, Nanjing, Jiangsu, China

4Department of Microbiology, Tumor and Cell Biology, Karolinska Institutet, Stockholm, Sweden.

5Oujiang Laboratory (Zhejiang Lab for Regenerative Medicine, Vison and Brain Health), School of Pharmaceutical Science, Wenzhou Medical University, Wenzhou, P.R. China.

6Longyan First Hospital Affiliated to Fujian Medical University, Longyan 364000, Fujian, China

7Department of Pathology, Shanghai Pulmonary Hospital, Tongji University School of Medicine, Shanghai, PR China

8Institute of Translational Medicine, Shanghai University, 99 Shangda Road, Shanghai 200444, China

9Department of Oral Implantology, School and Hospital of Stomatology, Tongji University; Shanghai Engineering Research Center of Tooth Restoration and Regeneration, Shanghai, China

Thank you so much for your help.

Best regards.

Yunlong Yang

Fudan University

Subject:	Re: CDDIS-22-0531RR authorship confirmation	
From:	"谢思思" <18111010026@fudan.edu.cn>	Aug 4, 2022 10:20:46 PM
To:	"Yunlong Yang" <yunlongyang@fudan.edu.cn>	

I agree with the above mentioned authorship.

Sisi Xie

-----原始邮件-----
发件人:"Yunlong Yang" <yunlongyang@fudan.edu.cn>
发送时间:2022-08-04 22:18:49 (星期四)
收件人: "孙琪" <19111010006@fudan.edu.cn>, "王雨洁" <wang_maggie_223@163.com>, dr_jihong@163.com, xiaoting <xiaoting9203@126.com>, "谢思思" <18111010026@fudan.edu.cn>, ltchen@163.com, "李森" <20211230014@fudan.edu.cn>, "曾苇凡" <17301010015@fudan.edu.cn>, "陈瑞波" <17301010009@fudan.edu.cn>, 15301050249@fudan.edu.cn, "左伋" <jzuo@shmu.edu.cn>, hlk9575@163.com, "Kayoko.Hosaka" <Kayoko.Hosaka@ki.se>, "卢永田" <luyongtian@263.net>, liuchanger1984@163.com, "叶颖" <ying.ye@tongji.edu.cn>
抄送:
主题: CDDIS-22-0531RR authorship confirmation

Dear co-authors,

Before our CDDIS paper is officially accepted, I need you to send an official confirmation of the current authorship.

Please reply to this email indicating you agree with the current authorship.

e.g. "I agree with the above mentioned authorship.

Your name in English"

Current authorship:

Qi Sun1#, Yujie Wang2#, Hong Ji3#, Xiaoting Sun1,4,5, Sisi Xie1,6, Longtian Chen6, Sen Li1, Weifan Zeng1, Ruibo Chen1, Qi Tang1, Ji Zuo1, Likun Hou7, Kayoko Hosaka4 Yongtian Lu2, Ying Liu8*, Ying Ye9* and Yunlong Yang1*

1Department of Cellular and Genetic Medicine, School of Basic Medical Sciences, Fudan University, Shanghai 200032, China

2Department of Otolaryngology, Shenzhen Key Laboratory of Nanozymes and Translational Cancer Research, Shenzhen Institute of Translational Medicine, The First Affiliated Hospital of Shenzhen University, Shenzhen Second People's Hospital, Shenzhen, Guangdong 518035, China

3Department of Radiation Oncology, Jiangsu Cancer Hospital & Jiangsu Institute of Cancer Research & The Affiliated Cancer Hospital of Nanjing Medical University, Nanjing, Jiangsu, China

4Department of Microbiology, Tumor and Cell Biology, Karolinska Institutet, Stockholm, Sweden.

5Oujiang Laboratory (Zhejiang Lab for Regenerative Medicine, Vison and Brain Health), School of Pharmaceutical Science, Wenzhou Medical University, Wenzhou, P.R. China.

6Longyan First Hospital Affiliated to Fujian Medical University, Longyan 364000, Fujian, China

7Department of Pathology, Shanghai Pulmonary Hospital, Tongji University School of Medicine, Shanghai, PR China

8Institute of Translational Medicine, Shanghai University, 99 Shangda Road, Shanghai 200444, China

9Department of Oral Implantology, School and Hospital of Stomatology, Tongji University; Shanghai Engineering Research Center of Tooth Restoration and Regeneration, Shanghai, China

Thank you so much for your help.

Best regards.

Yunlong Yang

Fudan University

Subject:	Re:CDDIS-22-0531RR authorship confirmation
From:	"陈隆天" <18906975165@163.com> Aug 4, 2022 10:41:36 PM
To:	"Yunlong Yang" <yunlongyang@fudan.edu.cn>
Cc:	18111010026@fudan.edu.cn

I agree with the above mentioned authorship.

Longtian Chen

Thank you.

At 2022-08-04 22:18:49, "Yunlong Yang" <yunlongyang@fudan.edu.cn> wrote:

> Dear co-authors,
>
> Before our CDDIS paper is officially accepted, I need you to send an official confirmation of the current authorship.
>
> Please reply to this email indicating you agree with the current authorship.
>
> e.g. "I agree with the above mentioned authorship.
>
> Your name in English"
>
> Current authorship:
>
> Qi Sun1#, Yujie Wang2#, Hong Ji3#, Xiaoting Sun1,4,5, Sisi Xie1,6, Longtian Chen6, Sen Li1, Weifan Zeng1, Ruibo Chen1, Qi Tang1, Ji Zuo1, Likun Hou7, Kayoko Hosaka4 Yongtian Lu2, Ying Liu8*, Ying Ye9* and Yunlong Yang1*
>
> 1Department of Cellular and Genetic Medicine, School of Basic Medical Sciences, Fudan University, Shanghai 200032, China
>
> 2Department of Otolaryngology, Shenzhen Key Laboratory of Nanozymes and Translational Cancer Research, Shenzhen Institute of Translational Medicine, The First Affiliated Hospital of Shenzhen University, Shenzhen Second People’s Hospital, Shenzhen, Guangdong 518035, China
>
> 3Department of Radiation Oncology, Jiangsu Cancer Hospital & Jiangsu Institute of Cancer Research & The Affiliated Cancer Hospital of Nanjing Medical University, Nanjing, Jiangsu, China

4Department of Microbiology, Tumor and Cell Biology, Karolinska Institutet, Stockholm, Sweden.

5Oujiang Laboratory (Zhejiang Lab for Regenerative Medicine, Vison and Brain Health), School of Pharmaceutical Science, Wenzhou Medical University, Wenzhou, P.R. China.

6Longyan First Hospital Affiliated to Fujian Medical University, Longyan 364000, Fujian, China

7Department of Pathology, Shanghai Pulmonary Hospital, Tongji University School of Medicine, Shanghai, PR China

8Institute of Translational Medicine, Shanghai University, 99 Shangda Road, Shanghai 200444, China

9Department of Oral Implantology, School and Hospital of Stomatology, Tongji University; Shanghai Engineering Research Center of Tooth Restoration and Regeneration, Shanghai, China

Thank you so much for your help.

Best regards.

Yunlong Yang

Fudan University

Subject:	Re: CDDIS-22-0531RR authorship confirmation	
From:	20211230014 <20211230014@fudan.edu.cn>	Aug 4, 2022 10:23:51 PM
To:	"Yunlong Yang" <yunlongyang@fudan.edu.cn>	

I agree with the above mentioned authorship.

Sen Li
在 2022年8月4日 下午10:18，Yunlong Yang <yunlongyang@fudan.edu.cn>写道：

> Dear co-authors,
>
> Before our CDDIS paper is officially accepted, I need you to send an official confirmation of the current authorship.
>
> Please reply to this email indicating you agree with the current authorship.
>
> e.g. "I agree with the above mentioned authorship.
>
> Your name in English"
>
> Current authorship:
>
> Qi Sun1#, Yujie Wang2#, Hong Ji3#, Xiaoting Sun1,4,5, Sisi Xie1,6, Longtian Chen6, Sen Li1, Weifan Zeng1, Ruibo Chen1, Qi Tang1, Ji Zuo1, Likun Hou7, Kayoko Hosaka4 Yongtian Lu2, Ying Liu8*, Ying Ye9* and Yunlong Yang1*
>
> 1Department of Cellular and Genetic Medicine, School of Basic Medical Sciences, Fudan University, Shanghai 200032, China
>
> 2Department of Otolaryngology, Shenzhen Key Laboratory of Nanozymes and Translational Cancer Research, Shenzhen Institute of Translational Medicine, The First Affiliated Hospital of Shenzhen University, Shenzhen Second People's Hospital, Shenzhen, Guangdong 518035, China
>
> 3Department of Radiation Oncology, Jiangsu Cancer Hospital & Jiangsu Institute of Cancer Research & The Affiliated Cancer Hospital of Nanjing Medical University, Nanjing, Jiangsu, China
>
> 4Department of Microbiology, Tumor and Cell Biology, Karolinska Institutet, Stockholm, Sweden.
>
> 5Oujiang Laboratory (Zhejiang Lab for Regenerative Medicine, Vison and Brain Health), School of Pharmaceutical Science, Wenzhou Medical University, Wenzhou, P.R. China.
>
> 6Longyan First Hospital Affiliated to Fujian Medical University, Longyan 364000, Fujian, China
>
> 7Department of Pathology, Shanghai Pulmonary Hospital, Tongji University School of Medicine, Shanghai, PR China
>
> 8Institute of Translational Medicine, Shanghai University, 99 Shangda Road, Shanghai 200444, China
>
> 9Department of Oral Implantology, School and Hospital of Stomatology, Tongji University; Shanghai Engineering Research Center of Tooth Restoration and Regeneration, Shanghai, China
>
> Thank you so much for your help.
>
> Best regards.
>
> Yunlong Yang
>
> Fudan University

Subject:	Re: CDDIS-22-0531RR authorship confirmation	
From:	"曾苇凡" <17301010015@fudan.edu.cn>	Aug 4, 2022 10:25:05 PM
To:	"Yunlong Yang" <yunlongyang@fudan.edu.cn>	

I agree with the above mentioned authorship.

Weifan Zeng

-----原始邮件-----
发件人:"Yunlong Yang" <yunlongyang@fudan.edu.cn>
发送时间:2022-08-04 22:18:49 (星期四)
收件人: "孙琪" <19111010006@fudan.edu.cn>, "王雨洁" <wang_maggie_223@163.com>, dr_jihong@163.com, xiaoting <xiaoting9203@126.com>, "谢思思" <18111010026@fudan.edu.cn>, ltchen@163.com, "李森" <20211230014@fudan.edu.cn>, "曾苇凡" <17301010015@fudan.edu.cn>, "陈瑞波" <17301010009@fudan.edu.cn>, 15301050249@fudan.edu.cn, "左伋" <jzuo@shmu.edu.cn>, hlk9575@163.com, "Kayoko.Hosaka" <Kayoko.Hosaka@ki.se>, "卢永田" <luyongtian@263.net>, liuchanger1984@163.com, "叶颖" <ying.ye@tongji.edu.cn>
抄送:
主题: CDDIS-22-0531RR authorship confirmation

Dear co-authors,

Before our CDDIS paper is officially accepted, I need you to send an official confirmation of the current authorship.

Please reply to this email indicating you agree with the current authorship.

e.g. "I agree with the above mentioned authorship.

Your name in English"

Current authorship:

Qi Sun1#, Yujie Wang2#, Hong Ji3#, Xiaoting Sun1,4,5, Sisi Xie1,6, Longtian Chen6, Sen Li1, Weifan Zeng1, Ruibo Chen1, Qi Tang1, Ji Zuo1, Likun Hou7, Kayoko Hosaka4 Yongtian Lu2, Ying Liu8*, Ying Ye9* and Yunlong Yang1*

1Department of Cellular and Genetic Medicine, School of Basic Medical Sciences, Fudan University, Shanghai 200032, China

2Department of Otolaryngology, Shenzhen Key Laboratory of Nanozymes and Translational Cancer Research, Shenzhen Institute of Translational Medicine, The First Affiliated Hospital of Shenzhen University, Shenzhen Second People's Hospital, Shenzhen, Guangdong 518035, China

3Department of Radiation Oncology, Jiangsu Cancer Hospital & Jiangsu Institute of Cancer Research & The Affiliated Cancer Hospital of Nanjing Medical University, Nanjing, Jiangsu, China

4Department of Microbiology, Tumor and Cell Biology, Karolinska Institutet, Stockholm, Sweden.

5Oujiang Laboratory (Zhejiang Lab for Regenerative Medicine, Vison and Brain Health), School of Pharmaceutical Science, Wenzhou Medical University, Wenzhou, P.R. China.

6Longyan First Hospital Affiliated to Fujian Medical University, Longyan 364000, Fujian, China

7Department of Pathology, Shanghai Pulmonary Hospital, Tongji University School of Medicine, Shanghai, PR China

8Institute of Translational Medicine, Shanghai University, 99 Shangda Road, Shanghai 200444, China

9Department of Oral Implantology, School and Hospital of Stomatology, Tongji University; Shanghai Engineering Research Center of Tooth Restoration and Regeneration, Shanghai, China

Thank you so much for your help.

Best regards.

Yunlong Yang

Fudan University

Subject:	Re: CDDIS-22-0531RR authorship confirmation	
From:	"陈瑞波" <17301010009@fudan.edu.cn>	Aug 4, 2022 10:25:12 PM
To:	"Yunlong Yang" <yunlongyang@fudan.edu.cn>	

I agree with the above mentioned authorship.

Ruibo Chen

-----原始邮件-----
发件人:"Yunlong Yang" <yunlongyang@fudan.edu.cn>
发送时间:2022-08-04 22:18:49 (星期四)
收件人: "孙琪" <19111010006@fudan.edu.cn>, "王雨洁" <wang_maggie_223@163.com>, dr_jihong@163.com, xiaoting <xiaoting9203@126.com>, "谢思思" <18111010026@fudan.edu.cn>, ltchen@163.com, "李森" <20211230014@fudan.edu.cn>, "曾苇凡" <17301010015@fudan.edu.cn>, "陈瑞波" <17301010009@fudan.edu.cn>, 15301050249@fudan.edu.cn, "左伋" <jzuo@shmu.edu.cn>, hlk9575@163.com, "Kayoko.Hosaka" <Kayoko.Hosaka@ki.se>, "卢永田" <luyongtian@263.net>, liuchanger1984@163.com, "叶颖" <ying.ye@tongji.edu.cn>
抄送:
主题: CDDIS-22-0531RR authorship confirmation

Dear co-authors,

Before our CDDIS paper is officially accepted, I need you to send an official confirmation of the current authorship.

Please reply to this email indicating you agree with the current authorship.

e.g. "I agree with the above mentioned authorship.

Your name in English"

Current authorship:

Qi Sun1#, Yujie Wang2#, Hong Ji3#, Xiaoting Sun1,4,5, Sisi Xie1,6, Longtian Chen6, Sen Li1, Weifan Zeng1, Ruibo Chen1, Qi Tang1, Ji Zuo1, Likun Hou7, Kayoko Hosaka4 Yongtian Lu2, Ying Liu8*, Ying Ye9* and Yunlong Yang1*

1Department of Cellular and Genetic Medicine, School of Basic Medical Sciences, Fudan University, Shanghai 200032, China

2Department of Otolaryngology, Shenzhen Key Laboratory of Nanozymes and Translational Cancer Research, Shenzhen Institute of Translational Medicine, The First Affiliated Hospital of Shenzhen University, Shenzhen Second People's Hospital, Shenzhen, Guangdong 518035, China

3Department of Radiation Oncology, Jiangsu Cancer Hospital & Jiangsu Institute of Cancer Research & The Affiliated Cancer Hospital of Nanjing Medical University, Nanjing, Jiangsu, China

4Department of Microbiology, Tumor and Cell Biology, Karolinska Institutet, Stockholm, Sweden.

5Oujiang Laboratory (Zhejiang Lab for Regenerative Medicine, Vison and Brain Health), School of Pharmaceutical Science, Wenzhou Medical University, Wenzhou, P.R. China.

6Longyan First Hospital Affiliated to Fujian Medical University, Longyan 364000, Fujian, China

7Department of Pathology, Shanghai Pulmonary Hospital, Tongji University School of Medicine, Shanghai, PR China

8Institute of Translational Medicine, Shanghai University, 99 Shangda Road, Shanghai 200444, China

9Department of Oral Implantology, School and Hospital of Stomatology, Tongji University; Shanghai Engineering Research Center of Tooth Restoration and Regeneration, Shanghai, China

Thank you so much for your help.

Best regards.

Yunlong Yang

Fudan University

Subject:	Re:CDDIS-22-0531RR authorship confirmation	
From:	"唐齐" <15301050249@fudan.edu.cn>	Aug 4, 2022 10:31:18 PM
To:	"Yunlong Yang" <yunlongyang@fudan.edu.cn>	
Cc:	"孙琪" <19111010006@fudan.edu.cn>, "王雨洁" <wang_maggie_223@163.com>, dr_jihong <dr_jihong@163.com>, xiaoting <xiaoting9203@126.com>, "谢思思" <18111010026@fudan.edu.cn>, ltchen <ltchen@163.com>, "李森" <20211230014@fudan.edu.cn>, "曾苇凡" <17301010015@fudan.edu.cn>, "陈瑞波" <17301010009@fudan.edu.cn>, "左伋" <jzuo@shmu.edu.cn>, hlk9575 <hlk9575@163.com>, "Kayoko.Hosaka" <Kayoko.Hosaka@ki.se>, "卢永田" <luyongtian@263.net>, liuchanger1984 <liuchanger1984@163.com>, "叶颖" <ying.ye@tongji.edu.cn>	

I agree with the above mentioned authorship.
Qi Tang

在 2022-08-04 22:18:49，"Yunlong Yang" <yunlongyang@fudan.edu.cn> 写道：

>Dear co-authors,
>
>
>Before our CDDIS paper is officially accepted, I need you to send an official confirmation of the current authorship.
>
>
>Please reply to this email indicating you agree with the current authorship.
>
>
>e.g. "I agree with the above mentioned authorship.
>
>
>Your name in English"
>
>
>
>Current authorship:
>
>
>Qi Sun1#, Yujie Wang2#, Hong Ji3#, Xiaoting Sun1,4,5, Sisi Xie1,6, Longtian Chen6, Sen Li1, Weifan Zeng1, Ruibo Chen1, Qi Tang1, Ji Zuo1, Likun Hou7, Kayoko Hosaka4 Yongtian Lu2, Ying Liu8*, Ying Ye9* and Yunlong Yang1*
>
>
>1Department of Cellular and Genetic Medicine, School of Basic Medical Sciences, Fudan University, Shanghai 200032, China
>
>
>2Department of Otolaryngology, Shenzhen Key Laboratory of Nanozymes and Translational Cancer Research, Shenzhen Institute of Translational Medicine, The First Affiliated Hospital of Shenzhen University, Shenzhen Second People’s Hospital, Shenzhen, Guangdong 518035, China
>
>
>3Department of Radiation Oncology, Jiangsu Cancer Hospital & Jiangsu Institute of Cancer Research & The Affiliated Cancer Hospital of Nanjing Medical University, Nanjing, Jiangsu, China
>
>
>4Department of Microbiology, Tumor and Cell Biology, Karolinska Institutet, Stockholm, Sweden.
>
>
>5Oujiang Laboratory (Zhejiang Lab for Regenerative Medicine, Vison and Brain Health), School of Pharmaceutical Science, Wenzhou Medical University, Wenzhou, P.R. China.
>
>
>6Longyan First Hospital Affiliated to Fujian Medical University, Longyan 364000, Fujian, China
>
>
>7Department of Pathology, Shanghai Pulmonary Hospital, Tongji University School of Medicine, Shanghai, PR China
>
>
>8Institute of Translational Medicine, Shanghai University, 99 Shangda Road, Shanghai 200444, China
>
>
>9Department of Oral Implantology, School and Hospital of Stomatology, Tongji University; Shanghai Engineering Research Center of Tooth Restoration and Regeneration, Shanghai, China
>

>
>
>
>Thank you so much for your help.
>
>
>Best regards.
>
>
>
>
>Yunlong Yang
>
>
>Fudan University
>
>
>
>
>
>
>
>

Subject:	Re: CDDIS-22-0531RR authorship confirmation
From:	"左伋" <jzuo@shmu.edu.cn> Aug 5, 2022 3:31:50 PM
To:	"Yunlong Yang" <yunlongyang@fudan.edu.cn>

I agree with the above mentioned authorship.

JI ZUO

-----原始邮件-----
发件人:"Yunlong Yang" <yunlongyang@fudan.edu.cn>
发送时间:2022-08-04 22:18:49 (星期四)
收件人: "孙琪" <19111010006@fudan.edu.cn>, "王雨洁" <wang_maggie_223@163.com>, dr_jihong@163.com, xiaoting <xiaoting9203@126.com>, "谢思思" <18111010026@fudan.edu.cn>, ltchen@163.com, "李森" <20211230014@fudan.edu.cn>, "曾苇凡" <17301010015@fudan.edu.cn>, "陈瑞波" <17301010009@fudan.edu.cn>, 15301050249@fudan.edu.cn, "左伋" <jzuo@shmu.edu.cn>, hlk9575@163.com, "Kayoko.Hosaka" <Kayoko.Hosaka@ki.se>, "卢永田" <luyongtian@263.net>, liuchanger1984@163.com, "叶颖" <ying.ye@tongji.edu.cn>
抄送:
主题: CDDIS-22-0531RR authorship confirmation

Dear co-authors,

Before our CDDIS paper is officially accepted, I need you to send an official confirmation of the current authorship.

Please reply to this email indicating you agree with the current authorship.

e.g. "I agree with the above mentioned authorship.

Your name in English"

Current authorship:

Qi Sun1#, Yujie Wang2#, Hong Ji3#, Xiaoting Sun1,4,5, Sisi Xie1,6, Longtian Chen6, Sen Li1, Weifan Zeng1, Ruibo Chen1, Qi Tang1, Ji Zuo1, Likun Hou7, Kayoko Hosaka4 Yongtian Lu2, Ying Liu8*, Ying Ye9* and Yunlong Yang1*

1Department of Cellular and Genetic Medicine, School of Basic Medical Sciences, Fudan University, Shanghai 200032, China

2Department of Otolaryngology, Shenzhen Key Laboratory of Nanozymes and Translational Cancer Research, Shenzhen Institute of Translational Medicine, The First Affiliated Hospital of Shenzhen University, Shenzhen Second People's Hospital, Shenzhen, Guangdong 518035, China

3Department of Radiation Oncology, Jiangsu Cancer Hospital & Jiangsu Institute of Cancer Research & The Affiliated Cancer Hospital of Nanjing Medical University, Nanjing, Jiangsu, China

4Department of Microbiology, Tumor and Cell Biology, Karolinska Institutet, Stockholm, Sweden.

5Oujiang Laboratory (Zhejiang Lab for Regenerative Medicine, Vison and Brain Health), School of Pharmaceutical Science, Wenzhou Medical University, Wenzhou, P.R. China.

6Longyan First Hospital Affiliated to Fujian Medical University, Longyan 364000, Fujian, China

7Department of Pathology, Shanghai Pulmonary Hospital, Tongji University School of Medicine, Shanghai, PR China

8Institute of Translational Medicine, Shanghai University, 99 Shangda Road, Shanghai 200444, China

9Department of Oral Implantology, School and Hospital of Stomatology, Tongji University; Shanghai Engineering Research Center of Tooth Restoration and Regeneration, Shanghai, China

Thank you so much for your help.

Best regards.

Yunlong Yang

Fudan University

Subject:	Re:CDDIS-22-0531RR authorship confirmation
From:	"侯立坤" <hlk9575@163.com> Aug 4, 2022 11:17:50 PM
To:	"Yunlong Yang" <yunlongyang@fudan.edu.cn>

I agree with the above mentioned authorship.

Likun Hou

At 2022-08-04 22:18:49, "Yunlong Yang" <yunlongyang@fudan.edu.cn> wrote:

Dear co-authors,

Before our CDDIS paper is officially accepted, I need you to send an official confirmation of the current authorship.

Please reply to this email indicating you agree with the current authorship.

e.g. "I agree with the above mentioned authorship.

Your name in English"

Current authorship:

Qi Sun1#, Yujie Wang2#, Hong Ji3#, Xiaoting Sun1,4,5, Sisi Xie1,6, Longtian Chen6, Sen Li1, Weifan Zeng1, Ruibo Chen1, Qi Tang1, Ji Zuo1, Likun Hou7, Kayoko Hosaka4 Yongtian Lu2, Ying Liu8*, Ying Ye9* and Yunlong Yang1*

1Department of Cellular and Genetic Medicine, School of Basic Medical Sciences, Fudan University, Shanghai 200032, China

2Department of Otolaryngology, Shenzhen Key Laboratory of Nanozymes and Translational Cancer Research, Shenzhen Institute of Translational Medicine, The First Affiliated Hospital of Shenzhen University, Shenzhen Second People's Hospital, Shenzhen, Guangdong 518035, China

3Department of Radiation Oncology, Jiangsu Cancer Hospital & Jiangsu Institute of Cancer Research & The Affiliated Cancer Hospital of Nanjing Medical University, Nanjing, Jiangsu, China

4Department of Microbiology, Tumor and Cell Biology, Karolinska Institutet, Stockholm, Sweden.

5Oujiang Laboratory (Zhejiang Lab for Regenerative Medicine, Vison and Brain Health), School of Pharmaceutical Science, Wenzhou Medical University, Wenzhou, P.R. China.

6Longyan First Hospital Affiliated to Fujian Medical University, Longyan 364000, Fujian, China

7Department of Pathology, Shanghai Pulmonary Hospital, Tongji University School of Medicine, Shanghai, PR China

8Institute of Translational Medicine, Shanghai University, 99 Shangda Road, Shanghai 200444, China

9Department of Oral Implantology, School and Hospital of Stomatology, Tongji University; Shanghai Engineering Research Center of Tooth Restoration and Regeneration, Shanghai, China

Thank you so much for your help.

Best regards.

Yunlong Yang

Fudan University

Subject:	Re: CDDIS-22-0531RR authorship confirmation	
From:	"Kayoko Hosaka" <kayoko.hosaka@ki.se>	Aug 4, 2022 10:28:26 PM
To:	"mikelong mikelong" <yunlongyang@fudan.edu.cn>	

Dear Yunlong,

I agree with the below-mentioned authorship for this paper.

Best regards,
Kayoko

Kayoko Hosaka, M.D., PhD.
Group: Dr. Yihai Cao
Department of Microbiology, Tumor and Cell biology (MTC)
Karolinska Institutet, Solnavägen 9, Biomedicum 8C
171 65 Solna, Sweden
Telephone, office: +46 8 52486299
Fax, office: +46 8 331399
E-mail: Kayoko.Hosaka@ki.se, khosaka1@yahoo.co.jp

From: Yunlong Yang <yunlongyang@fudan.edu.cn>
Sent: Thursday, August 4, 2022 4:18 PM
To: 孙琪 <19111010006@fudan.edu.cn>; 王雨洁 <wang_maggie_223@163.com>; dr_jihong@163.com <dr_jihong@163.com>; xiaoting <xiaoting9203@126.com>; 谢思思 <18111010026@fudan.edu.cn>; ltchen@163.com <ltchen@163.com>; 李森 <20211230014@fudan.edu.cn>; 曾苇凡 <17301010015@fudan.edu.cn>; 陈瑞波 <17301010009@fudan.edu.cn>; 15301050249@fudan.edu.cn <15301050249@fudan.edu.cn>; 左伋 <jzuo@shmu.edu.cn>; hlk9575@163.com <hlk9575@163.com>; Kayoko Hosaka <kayoko.hosaka@ki.se>; 卢永田 <luyongtian@263.net>; liuchanger1984@163.com <liuchanger1984@163.com>; 叶颖 <ying.ye@tongji.edu.cn>
Subject: CDDIS-22-0531RR authorship confirmation

Dear co-authors,

Before our CDDIS paper is officially accepted, I need you to send an official confirmation of the current authorship.

Please reply to this email indicating you agree with the current authorship.

e.g. "I agree with the above mentioned authorship.

Your name in English"

Current authorship:

Qi Sun1#, Yujie Wang2#, Hong Ji3#, Xiaoting Sun1,4,5, Sisi Xie1,6, Longtian Chen6, Sen Li1, Weifan Zeng1, Ruibo Chen1, Qi Tang1, Ji Zuo1, Likun Hou7, Kayoko Hosaka4 Yongtian Lu2, Ying Liu8*, Ying Ye9* and Yunlong Yang1*

1Department of Cellular and Genetic Medicine, School of Basic Medical Sciences, Fudan University, Shanghai 200032, China

2Department of Otolaryngology, Shenzhen Key Laboratory of Nanozymes and Translational Cancer Research, Shenzhen Institute of Translational Medicine, The First Affiliated Hospital of Shenzhen University, Shenzhen Second People's Hospital, Shenzhen, Guangdong 518035, China

3Department of Radiation Oncology, Jiangsu Cancer Hospital & Jiangsu Institute of Cancer Research & The Affiliated Cancer Hospital of Nanjing Medical University, Nanjing, Jiangsu, China

4Department of Microbiology, Tumor and Cell Biology, Karolinska Institutet, Stockholm, Sweden.

5Oujiang Laboratory (Zhejiang Lab for Regenerative Medicine, Vison and Brain Health), School of Pharmaceutical Science, Wenzhou Medical University, Wenzhou, P.R. China.

6Longyan First Hospital Affiliated to Fujian Medical University, Longyan 364000, Fujian, China

7Department of Pathology, Shanghai Pulmonary Hospital, Tongji University School of Medicine, Shanghai, PR China

8Institute of Translational Medicine, Shanghai University, 99 Shangda Road, Shanghai 200444, China

9Department of Oral Implantology, School and Hospital of Stomatology, Tongji University; Shanghai Engineering Research Center of Tooth Restoration and Regeneration, Shanghai, China

Thank you so much for your help.

Best regards.

Yunlong Yang

Fudan University

När du skickar e-post till Karolinska Institutet (KI) innebär detta att KI kommer att behandla dina personuppgifter. Här finns information om hur KI behandlar personuppgifter.

Sending email to Karolinska Institutet (KI) will result in KI processing your personal data. You can read more about KI's processing of personal data here.

Subject:	Re:CDDIS-22-0531RR authorship confirmation
From:	"卢永田" <luyongtian@263.net> Aug 5, 2022 2:41:03 PM
To:	"Yunlong Yang" <yunlongyang@fudan.edu.cn>, "孙琪 " <19111010006@fudan.edu.cn>, "王雨洁 " <wang_maggie_223@163.com>, dr_jihong <dr_jihong@163.com>, xiaoting <xiaoting9203@126.com>, "谢思思 " <18111010026@fudan.edu.cn>, ltchen <ltchen@163.com>, "李森 " <20211230014@fudan.edu.cn>, "曾苇凡 " <17301010015@fudan.edu.cn>, "陈瑞波 " <17301010009@fudan.edu.cn>, 15301050249 <15301050249@fudan.edu.cn>, "左伋 " <jzuo@shmu.edu.cn>, hlk9575 <hlk9575@163.com>, "Kayoko.Hosaka " <kayoko.hosaka@ki.se>, "卢永田 " <luyongtian@263.net>, liuchanger1984 <liuchanger1984@163.com>, "叶颖 " <ying.ye@tongji.edu.cn>

I agree with the above mentioned authorship. Yongtian Lu

----- Original Message -----
From:Yunlong Yang <yunlongyang@fudan.edu.cn>
To: 孙琪 <19111010006@fudan.edu.cn> 王雨洁 <wang_maggie_223@163.com> dr_jihong <dr_jihong@163.com> xiaoting <xiaoting9203@126.com> 谢思思 <18111010026@fudan.edu.cn> ltchen <ltchen@163.com> 李森 <20211230014@fudan.edu.cn> 曾苇凡 <17301010015@fudan.edu.cn> 陈瑞波 <17301010009@fudan.edu.cn> 15301050249 <15301050249@fudan.edu.cn> 左伋 <jzuo@shmu.edu.cn> hlk9575 <hlk9575@163.com> Kayoko.Hosaka <Kayoko.Hosaka@ki.se> 卢永田 <luyongtian@263.net> liuchanger1984 <liuchanger1984@163.com> 叶颖 <ying.ye@tongji.edu.cn>
CC:
Sent: 2022-08-04 22:18
Subject: Re:CDDIS-22-0531RR authorship confirmation

Dear co-authors,

Before our CDDIS paper is officially accepted, I need you to send an official confirmation of the current authorship.

Please reply to this email indicating you agree with the current authorship.

e.g. "I agree with the above mentioned authorship.

Your name in English"

Current authorship:

Qi Sun1#, Yujie Wang2#, Hong Ji3#, Xiaoting Sun1,4,5, Sisi Xie1,6, Longtian Chen6, Sen Li1, Weifan Zeng1, Ruibo Chen1, Qi Tang1, Ji Zuo1, Likun Hou7, Kayoko Hosaka4 Yongtian Lu2, Ying Liu8*, Ying Ye9* and Yunlong Yang1*

1Department of Cellular and Genetic Medicine, School of Basic Medical Sciences, Fudan University, Shanghai 200032, China

2Department of Otolaryngology, Shenzhen Key Laboratory of Nanozymes and Translational Cancer Research, Shenzhen Institute of Translational Medicine, The First Affiliated Hospital of Shenzhen University, Shenzhen Second People’s Hospital, Shenzhen, Guangdong 518035, China

3Department of Radiation Oncology, Jiangsu Cancer Hospital & Jiangsu Institute of Cancer Research & The Affiliated Cancer Hospital of Nanjing Medical University, Nanjing, Jiangsu, China

4Department of Microbiology, Tumor and Cell Biology, Karolinska Institutet, Stockholm, Sweden.

5Oujiang Laboratory (Zhejiang Lab for Regenerative Medicine, Vison and Brain Health), School of Pharmaceutical Science, Wenzhou Medical University, Wenzhou, P.R. China.

6Longyan First Hospital Affiliated to Fujian Medical University, Longyan 364000, Fujian, China

7Department of Pathology, Shanghai Pulmonary Hospital, Tongji University School of Medicine, Shanghai, PR China

8Institute of Translational Medicine, Shanghai University, 99 Shangda Road, Shanghai 200444, China

9Department of Oral Implantology, School and Hospital of Stomatology, Tongji University; Shanghai Engineering Research Center of Tooth Restoration and Regeneration, Shanghai, China

Thank you so much for your help.

Best regards.

Yunlong Yang

Fudan University

Subject:	Re:CDDIS-22-0531RR authorship confirmation
From:	"刘莹" <liuchanger1984@163.com> Aug 4, 2022 10:29:45 PM
To:	"Yunlong Yang" <yunlongyang@fudan.edu.cn>

I agree with the above mentioned authorship.

Ying Liu

At 2022-08-04 22:18:49, "Yunlong Yang" <yunlongyang@fudan.edu.cn> wrote:

> Dear co-authors,
>
> Before our CDDIS paper is officially accepted, I need you to send an official confirmation of the current authorship.
>
> Please reply to this email indicating you agree with the current authorship.
>
> e.g. "I agree with the above mentioned authorship.
>
> Your name in English"
>
> Current authorship:
>
> Qi Sun1#, Yujie Wang2#, Hong Ji3#, Xiaoting Sun1,4,5, Sisi Xie1,6, Longtian Chen6, Sen Li1, Weifan Zeng1, Ruibo Chen1, Qi Tang1, Ji Zuo1, Likun Hou7, Kayoko Hosaka4 Yongtian Lu2, Ying Liu8*, Ying Ye9* and Yunlong Yang1*
>
> 1Department of Cellular and Genetic Medicine, School of Basic Medical Sciences, Fudan University, Shanghai 200032, China
>
> 2Department of Otolaryngology, Shenzhen Key Laboratory of Nanozymes and Translational Cancer Research, Shenzhen Institute of Translational Medicine, The First Affiliated Hospital of Shenzhen University, Shenzhen Second People’s Hospital, Shenzhen, Guangdong 518035, China
>
> 3Department of Radiation Oncology, Jiangsu Cancer Hospital & Jiangsu Institute of Cancer Research & The Affiliated Cancer Hospital of Nanjing Medical University, Nanjing, Jiangsu, China
>
> 4Department of Microbiology, Tumor and Cell Biology, Karolinska Institutet, Stockholm, Sweden.

5Oujiang Laboratory (Zhejiang Lab for Regenerative Medicine, Vison and Brain Health), School of Pharmaceutical Science, Wenzhou Medical University, Wenzhou, P.R. China.

6Longyan First Hospital Affiliated to Fujian Medical University, Longyan 364000, Fujian, China

7Department of Pathology, Shanghai Pulmonary Hospital, Tongji University School of Medicine, Shanghai, PR China

8Institute of Translational Medicine, Shanghai University, 99 Shangda Road, Shanghai 200444, China

9Department of Oral Implantology, School and Hospital of Stomatology, Tongji University; Shanghai Engineering Research Center of Tooth Restoration and Regeneration, Shanghai, China

Thank you so much for your help.

Best regards.

Yunlong Yang

Fudan University

Subject:	Re: CDDIS-22-0531RR authorship confirmation
From:	"Ying Ye" <ying.ye@icloud.com> Aug 4, 2022 10:37:50 PM
To:	"Yunlong Yang" <yunlongyang@fudan.edu.cn>

I agree with the above mentioned authorship.
Ying Ye

My current affiliation has been changed into:

Department of Oral Implantology，Stomatological Hospital and Dental School of Tongji University, Shanghai Engineering Research Center of Tooth Restoration and Regeneration, Shanghai, China.

叶颖

在 2022年8月4日，22:10，Yunlong Yang <yunlongyang@fudan.edu.cn> 写道：

Dear co-authors,

Before our CDDIS paper is officially accepted, I need you to send an official confirmation of the current authorship.

Please reply to this email indicating you agree with the current authorship.

e.g. "I agree with the above mentioned authorship.

Your name in English"

Current authorship:

Qi Sun1#, Yujie Wang2#, Hong Ji3#, Xiaoting Sun1,4,5, Sisi Xie1,6, Longtian Chen6, Sen Li1, Weifan Zeng1, Ruibo Chen1, Qi Tang1, Ji Zuo1, Likun Hou7, Kayoko Hosaka4 Yongtian Lu2, Ying Liu8*, Ying Ye9* and Yunlong Yang1*

1Department of Cellular and Genetic Medicine, School of Basic Medical Sciences, Fudan University, Shanghai 200032, China

2Department of Otolaryngology, Shenzhen Key Laboratory of Nanozymes and Translational Cancer Research, Shenzhen Institute of Translational Medicine, The First Affiliated Hospital of Shenzhen University, Shenzhen Second People's Hospital, Shenzhen, Guangdong 518035, China

3Department of Radiation Oncology, Jiangsu Cancer Hospital & Jiangsu Institute of Cancer Research & The Affiliated Cancer Hospital of Nanjing Medical University, Nanjing, Jiangsu, China

4Department of Microbiology, Tumor and Cell Biology, Karolinska Institutet, Stockholm, Sweden.

5Oujiang Laboratory (Zhejiang Lab for Regenerative Medicine, Vison and Brain Health), School of Pharmaceutical Science, Wenzhou Medical University, Wenzhou, P.R. China.

6Longyan First Hospital Affiliated to Fujian Medical University, Longyan 364000, Fujian, China

7Department of Pathology, Shanghai Pulmonary Hospital, Tongji University School of Medicine, Shanghai, PR China

8Institute of Translational Medicine, Shanghai University, 99 Shangda Road, Shanghai 200444, China

9Department of Oral Implantology, School and Hospital of Stomatology, Tongji University; Shanghai Engineering Research Center of Tooth Restoration and Regeneration, Shanghai, China

Thank you so much for your help.

Best regards.

Yunlong Yang

Fudan University
